# Smartphone-Based Ecological Momentary Assessment of Pain in Older Adults Undergoing Auricular Point Acupressure for Chronic Low Back Pain: Secondary Analysis of a Randomized Controlled Trial

**DOI:** 10.2196/79612

**Published:** 2026-03-04

**Authors:** Nada Lukkahatai, Chitchanok Benjasirisan, Xinran Huang, Hulin Wu, Jennifer Kawi, Jingyu Zhang, Claudia M Campbell, Constance M Johnson, Paul J Christo, Johannes Thrul

**Affiliations:** 1 School of Nursing Johns Hopkins University Baltimore, MD United States; 2 Faculty of Nursing Mahidol University Bangkok, Siriraj Thailand; 3 School of Public Health University of Texas Health Science Center at Houston Houston, TX United States; 4 Cizik School of Nursing University of Texas Health Science Center at Houston Houston, TX United States; 5 School of Medicine Johns Hopkins University Baltimore, MD United States; 6 Department of Mental Health Bloomberg School of Public Health Johns Hopkins University Baltimore, MD United States

**Keywords:** ecological momentary assessment, auricular point acupressure, chronic low back pain, randomized controlled trial, pain

## Abstract

**Background:**

Chronic low back pain (cLBP) is a prevalent and disabling condition in older adults. Auricular point acupressure (APA), a nonpharmacologic intervention, has shown promise in managing cLBP. However, its impact on daily symptom fluctuations remains unclear. Ecological momentary assessment (EMA), which collects real-time data, offers a method to capture these fluctuations.

**Objective:**

This secondary analysis evaluated the effects of APA on EMA-reported pain intensity and pain interference among older adults with cLBP and examined associations among APA practice patterns, recall-based assessments, and EMA engagement.

**Methods:**

Data were drawn from a published 3-arm randomized controlled trial of 272 adults aged ≥60 years with cLBP, randomized to targeted auricular point acupressure (T-APA), nontargeted auricular point acupressure (NT-APA), or education control. For this analysis, only participants who completed at least one EMA entry during the 4-week intervention were included. A total of 61 participants were excluded due to missing EMA data, resulting in a final analytic sample of 211 (T-APA: 72; NT-APA: 74; and control: 65). EMA-reported pain intensity and interference were collected using a smartphone app 3 times daily over 29 days. Linear mixed-effects models assessed the effects of group assignment and APA practice behaviors on EMA outcomes, adjusting for demographics, smoking, opioid use, and baseline recall-based pain. Spearman correlations assessed associations between EMA and 7-day recall measures.

**Results:**

The overall compliance rate was 44.8%, with an attrition rate of 54%. There were no significant differences in compliance or attrition across treatment groups. Older participants showed significantly lower compliance, though attrition was not associated with age. Pain intensity reported via EMA was significantly lower than recall-based pain, while EMA-reported pain interference was higher. EMA and 7-day recall pain outcomes were strongly correlated (Spearman =0.53–0.95; *P*<.001). Both T-APA and NT-APA, analyzed as separate randomized groups, significantly reduced EMA-reported pain and interference compared to the control. T-APA reduced worst pain (β=–0.98, SE 0.33; *P*<.001), average pain (β=–0.93, SE 0.30; *P*<.001), and current pain (β=–1.01, SE 0.36; *P*=.006). NT-APA also reduced worst (β=–0.74, SE 0.12; *P*<.001), average (β=–1.02, SE 0.30; *P*=.001), and current pain (β=–1.26, SE 0.37; *P*=.001). For interference, T-APA significantly reduced interference with enjoyment of life (β=–1.72, SE 0.37; *P*<.001) and daily activity (β=–1.41, SE 0.34; *P*=.001), with similar reductions seen in NT-APA.

**Conclusions:**

APA significantly reduced daily pain and interference among older adults with cLBP. EMA provided valuable insights into treatment response and symptom variability. Future research should enhance EMA adherence and explore sustained APA use for self-management.

**Trial Registration:**

ClinicalTrials.gov NCT03589703; https://clinicaltrials.gov/study/NCT03589703

**International Registered Report Identifier (IRRID):**

RR2-10.1186/s13063-019-4016-x

## Introduction

Chronic low back pain (cLBP) is a common and debilitating condition affecting approximately 20.5% of older adults in the United States [1]. It contributes to substantial physical disability, reduced quality of life, and elevated risks of depression and social isolation [2]. As pharmacologic treatments may pose safety risks in this population, there is growing interest in nonpharmacologic approaches that support self-management [3,4]. Auricular point acupressure (APA), a low-cost and noninvasive technique rooted in traditional Chinese medicine, involves applying small seeds to specific points on the ear corresponding to body regions to relieve pain. APA is safe, easy to administer, and can be integrated into daily routines. To evaluate its effectiveness, we conducted a 3-arm randomized controlled trial (RCT) of 272 older adults with cLBP. Participants were randomized to receive either targeted auricular point acupressure (T-APA; ear points related to low back), nontargeted auricular point acupressure (NT-APA; ear points unrelated to low back), or an educational control. The study demonstrated that both APA groups reported significantly greater improvements in self-reported pain intensity and physical function compared to the control group. Details of the study protocol and primary outcomes have been published previously [5,6].

While these findings support the clinical benefits of APA, the interpretation of self-reported outcomes is limited by reliance on retrospective patient-reported outcome measures, such as weekly or monthly recall of pain. These traditional assessments are susceptible to memory bias and may not capture the moment-to-moment variability of pain—particularly in older adults [7]. Chronic pain is increasingly recognized as a fluctuating and context-dependent experience, with substantial within-day and day-to-day variability that is poorly characterized by summary recall measures alone [8,9]. Ecological momentary assessment (EMA), which prompts participants to report symptoms in real time using smartphones, has been increasingly used in chronic pain research to address these limitations. EMA reduces recall bias, improves ecological validity, and enables the examination of intraindividual pain variability and symptom dynamics that are not captured by conventional patient-reported outcome measures [7,10]. EMA studies in chronic pain populations have demonstrated that pain intensity varies substantially within individuals, that such variability is stable over time yet heterogeneous across patients, and that variability metrics may be independently associated with quality of life, emotional functioning, and pain-related interference beyond mean pain levels [8,9,11].

For older adults, EMA may offer additional advantages by accommodating mobility limitations and supporting more accurate symptom reporting in daily life by addressing common challenges in pain assessment, such as memory lapses and difficulty recalling pain episodes. EMA has also been shown to be feasible and acceptable in chronic pain populations when thoughtfully designed, although adherence remains a challenge in studies requiring frequent prompts over extended periods [12,13]. Emerging work further suggests that EMA is uniquely suited to examine modifiable psychosocial and behavioral processes relevant to pain, including emotion regulation, pain catastrophizing, substance use, and treatment engagement, which may explain on a momentary timescale [9,14,15].

Despite growing use of EMA in chronic pain research, its application in evaluating nonpharmacologic, self-administered interventions, such as APA, remains limited. To assess the feasibility of integrating EMA into a pain management and APA trial, we conducted a pilot study involving a 4-week APA intervention among older adults with cLBP [12]. Among 18 participants, adherence to EMA prompts was high (87%), usability ratings were favorable, and there were significant positive correlations between EMA and recall-based pain scores. These findings supported the integration of EMA into the larger RCT to enhance symptom assessment accuracy and better capture real-time treatment responses.

Building on these pilot findings, we incorporated EMA into our larger RCT evaluating the effectiveness of APA for managing cLBP in older adults. The study protocol was published [5], and primary outcomes demonstrating significant improvements in pain and physical function were reported [6]. In addition to standard retrospective self-reported measures, the trial prospectively integrated EMA via a smartphone app to capture real-time pain experiences and monitor adherence to APA practice. This approach allowed for examination of both average treatment effects and daily symptom trajectories, addressing recent calls to move beyond static pain outcomes toward dynamic, patient-centered measures in chronic pain research [16,17].

The purpose of this secondary analysis was to examine EMA-reported pain outcomes collected via a smartphone-based (EarAPA) app (developed by Chi-Chih Chen) and explore the relationship between APA practice patterns and daily fluctuations in pain intensity and interference.

## Methods

### Design

This study is a secondary analysis of EMA data collected as part of a previously published RCT evaluating APA for older adults with cLBP. The parent study protocol and primary outcomes have been reported elsewhere [5,6]. The original trial was registered at ClinicalTrials.gov (NCT03589703). This analysis focused on examining real-time pain intensity and interference captured via EMA and exploring their associations with APA practice patterns. The study is reported in accordance with the CONSORT-EHEALTH (Consolidated Standards of Reporting Trials of Electronic and Mobile Health Applications and Online Telehealth) checklist (version 1.6.1) in Multimedia Appendix 1 [18].

### Participants

This secondary analysis used data from a previously published RCT that evaluated the effectiveness of APA for managing cLBP in older adults [5,6]. The parent trial enrolled 272 participants who were randomized to T-APA (n=92), NT-APA (n=91), or an education control group (n=89). Recruitment occurred in 2 phases, from March 1, 2019, to March 13, 2020, and from June 12, 2021, to November 18, 2021, with follow-up completed by December 1, 2022.

For this analysis, inclusion was limited to individuals who had at least one EMA data point during the 4-week intervention period. Because the analytic aim was to examine real-time pain outcomes and their associations with APA practice, 61 participants without EMA data were excluded. The final analytic sample consisted of 211 participants with 72 in the T-APA group, 74 in the NT-APA group, and 65 in the control group.

### Recruitment

This secondary analysis used data from a previously conducted RCT evaluating APA for cLBP in older adults. Recruitment procedures, eligibility criteria, and informed consent processes have been detailed in the published protocol [5] and primary outcomes paper. Participants were recruited from outpatient clinical sites (eg, pain management, geriatrics, and internal medicine), community outreach, an aging research registry, social media platforms (including Craigslist), and ClinicalTrials.gov.

### Randomization and Intervention

This secondary analysis used data from an RCT in which participants were originally assigned to one of 3 study arms in a 1:1:1 ratio: T-APA, NT-APA, or education control. Randomization procedures, including using a computer-generated allocation sequence and block randomization, are described in detail in the published protocol [5]. In the parent trial, participants in the APA arms were blinded to their group assignment, and blinding was maintained for investigators, data collectors, and analysts.

The APA intervention protocol is described previously [5]. Participants in the groups were instructed to apply small seeds to designated auricular points 3 times daily, pressing each point for 3 minutes per session. All auricular points were selected based on standardized Chinese ear acupuncture maps, which correspond to specific regions of the ear to particular body parts and organ systems. The T-APA group received acupressure at points associated with cLBP (eg, lower back, nervous subcortex, sympathetic, and Shenmen), while the NT-APA group received acupressure at auricular points that were strategically selected to be unrelated to the low back (eg, mouth, stomach, duodenum, internal ear, and tonsil) [19,20]. Although NT-APA was described as a placebo control in the original trial design, it involved active auricular stimulation and was therefore retained as a distinct intervention arm in this secondary analysis to reflect its procedural characteristics while maintaining consistency with the parent RCT. The education control group received a booklet outlining evidence-based strategies for managing chronic back pain. All participants attended weekly sessions over 4 weeks to receive new seeds (APA groups) or review educational materials (control). A smartphone app (EarAPA) was used to collect real-time pain data and monitor adherence to the APA protocol.

### Smartphone EMA (EarAPA App)

Participants received a study smartphone (HTC Desire 612; HTC Corporation) with the EarAPA app preinstalled, along with a charger. The EarAPA app, adapted from the previously developed Earpress app [12], originally delivered educational materials and symptom surveys. For this study, the app was further customized to support EMA and to enhance usability for older adults by incorporating features, such as adjustable volume and larger font size [21]. Although the app was available on Android and iOS platforms, access was restricted to enrolled participants.

This EarAPA app was used to deliver EMA prompts and surveys over a 29-day period to facilitate real-time symptom tracking. Two types of EMA surveys were administered: (1) random EMA, issued once daily during waking hours, included 4 brief questions assessing momentary pain intensity and interference with enjoyment of life and daily activities; and (2) time-contingent EMA, prompted twice daily based on participants’ routines, captured information on pain, APA adherence, and analgesic use. The random EMA surveys took approximately 1 minute to complete, while time-contingent surveys took less than 2 minutes. All data were automatically uploaded to a secure, cloud-based server in real time, allowing research staff to monitor participation and follow up as needed.

### Measurement

#### Baseline Characteristics

This analysis used data collected as part of the parent RCT of APA for cLBP in older adults. Baseline characteristics, including age, sex, race or ethnicity, education level, employment status, income, BMI, smoking status, and opioid use, were collected before intervention initiation.

#### Pain Measures

##### Recall-Based Pain Intensity and Interference

Recall-based pain intensity worst pain and average pain) and interference (overall pain interference with daily activity and overall pain interference with enjoyment in life) were assessed weekly using items from the Brief Pain Inventory–Short Form (BPI-SF). All items used a 0-10 numeric rating scale, where 0 indicated “no pain” or “does not interfere” and 10 indicated “pain as bad as you can imagine” or “completely interferes.” The BPI-SF has been validated in various populations and demonstrates strong internal consistency, with Cronbach α ranging from 0.82 to 0.95 [22].

##### EMA-Based Pain Intensity and Interference

Real-time pain intensity and pain interference were assessed using EMA surveys delivered via the EarAPA smartphone app. EMA assessments were administered 3 times daily over a 29-day period, allowing for repeated measurement of pain experiences in participants’ natural environments. During daytime assessments, current pain intensity was reported using a 0-10 numeric rating scale, where 0 indicated “no pain” and 10 indicated “pain as bad as you can imagine.” Evening assessments measured the worst and average pain experienced that day, as well as pain interference using the same 0-10 scale, with 0 indicating “does not interfere” and 10 indicating “completely interferes.”

Both recall-based pain and EMA-based pain assessments were collected concurrently during the intervention period to enable direct comparison between retrospective weekly reports and real-time, momentary pain outcomes within the same time frame.

#### APA Practice and Analgesic Use

Daily self-report of APA practice behaviors, including frequency of pressing the taped seeds, minutes per pressing, and total pressing time, was collected via the evening EMA survey. Participants also reported daily analgesic use, including medication name, dosage, and frequency of use, via evening EMA assessment.

### Analysis

Data from the EarAPA smartphone app were analyzed using our published EMA analytic methods [12]. The analytic window included EMA data collected during the first month of treatment, defined as days 0 through 28 following baseline assessment. Descriptive statistics were used to summarize demographic characteristics, EMA compliance, and attrition. EMA compliance was defined as the proportion of completed EMA assessments relative to the total number of scheduled assessments (87 assessments per participant: 29 days×3 times per day). EMA attrition was defined as discontinuation of EMA participation during the intervention period.

Two-tailed paired *t* tests were used to compare weekly mean EMA-reported pain intensity and pain interference with corresponding 7-day recall-based assessments. Longitudinal associations between intervention assignment, APA practice behaviors, and pain outcomes were examined using linear mixed-effects models with repeated EMA measures nested within individuals to account for within-person correlation over time. Fixed effects included treatment group (T-APA, NT-APA, and education control), APA practice variables (pressing frequency, minutes per pressing, and total pressing time), analgesic use, day since baseline, and relevant covariates (age, sex, race, smoking status, employment status, baseline opioid use, baseline pain measures, and pain interference ratings). Random effects included participant-specific intercepts and slopes for APA practice and analgesic use, with temporal autocorrelation modeled using a first-order autoregressive covariance structure. All inferential analyses preserved the 3 original randomized groups. The 2 APA arms were not pooled for any statistical comparisons. Linear mixed-effects models were selected instead of ANOVA to accommodate repeated, unequally spaced EMA measurements and varying numbers of assessments per participant.

Because EMA data are inherently unbalanced due to variable response patterns across participants and time points, mixed-effects models allowed inclusion of all available observations without imputing missing values under the missing-at-random assumption. Total daily pressing time was calculated as the product of daily pressing frequency and minutes per pressing and was treated as a time-varying predictor. The analytic sample size was determined by the number of participants with available EMA data from the parent RCT. No a priori sample size calculation was performed for this secondary analysis. All analyses were conducted using R version 4.3.1 (R Foundation for Statistical Computing).

### Ethical Considerations

The parent RCT [5,6] was approved by the Johns Hopkins Medicine Institutional Review Board (IRB00175409). This secondary analysis used data collected under this original approval and did not require additional ethics review because all data were deidentified prior to analysis. Informed consent was obtained before enrollment, including consent for the collection of real-time symptom data using a smartphone-based EMA app. A copy of the consent form is available in Multimedia Appendix 1, following CONSORT-EHEALTH recommendations. To ensure privacy and confidentiality, EMA and survey data were transmitted securely and stored on encrypted, password-protected servers accessible only to authorized research staff of the parent study, with all identifying information removed before secondary analyses were conducted. Compensation was provided as part of the parent trial in accordance with the approved protocol. No additional compensation was offered for this secondary analysis, which involved only previously collected data.

## Results

### Demographics and Baseline Characteristics

**Figure 1:**
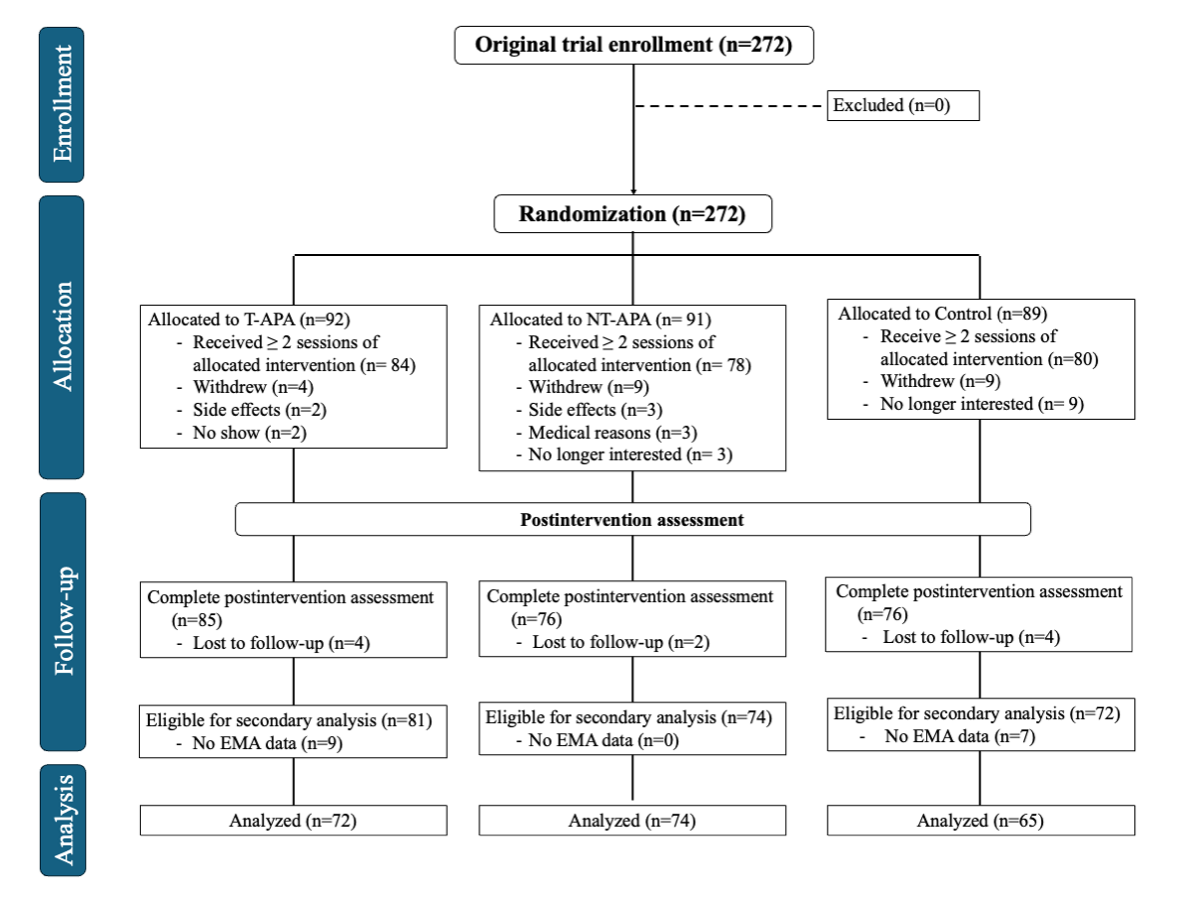
CONSORT (Consolidated Standards of Reporting Trials) diagram of the parent randomized controlled trial and eligibility for the ecological momentary assessment secondary analysis. EMA: ecological momentary assessment; NT-APA: nontargeted auricular point acupressure; T-APA: targeted auricular point acupressure.

This secondary analysis included 211 participants from a previously published RCT. As detailed in the Methods (Participants and Recruitment Sections), participants were included in this analysis if they had at least one EMA entry recorded during the 4-week intervention period. The final analytic sample comprised 72 participants in the T-APA group, 74 in the NT-APA group, and 65 in the education control group (Figure 1).

The analytic sample was generally balanced across the 3 study arms in terms of age, sex, race or ethnicity, education level, employment status, BMI, smoking status, and opioid use at baseline (Table 1). Overall, the sample had a mean age of 69.4 (SD 6.70) years and was predominantly female (135/211, 64%), with 63.5% (134/211) identifying as Black or African American. Most participants were retired or unemployed (181/211, 85.8%), and approximately 46% (97/211) reported current or recent opioid use.

**Table 1:** Demographics and baseline characteristics of all the participants who had ecological momentary assessment data (N=211).

Characteristic			T-APA^a^ (n=72)		NT-APA^b^ (n=74)		Control (n=65)		Overall (N=211)
Age (years), mean (SD; range)			69.6 (6.28; 60.0-87.0)		67.9 (6.93; 60.0-87.0)		70.7 (6.76; (60.0-91.0)		69.4 (6.70; 60.0-91.0)
**Sex, n (%)**									
	Female	46 (63.9)		46 (62.2)		43 (66.2)		135 (64)	
	Male	25 (34.7)		28 (37.8)		21 (32.3)		74 (35.1)	
	Unspecified	1 (1.4)		0 (0)		1 (1.5)		2 (0.9)	
**Race, n (%)**									
	American Indian or Alaska Native	0 (0)		0 (0)		2 (3.1)		2 (0.9)	
	Asian	0 (0)		1 (1.4)		0 (0)		1 (0.5)	
	Black or African American	41 (56.9)		49 (66.2)		44 (67.7)		134 (63.5)	
	White	29 (40.3)		21 (28.4)		18 (27.7)		68 (32.2)	
	Native Hawaiian or Pacific Islander	0 (0)		1 (1.4)		0 (0)		1 (0.5)	
	More than one race	1 (1.4)		2 (2.7)		0 (0)		3 (1.4)	
	Unknown or not reported	1 (1.4)		0 (0)		1 (1.5)		2 (1)	
**Ethnicity, n (%)**									
	Hispanic or Latino	1 (1.4)		0 (0)		2 (3.1)		3 (1.4)	
	Non-Hispanic or Latino	66 (91.7)		57 (77)		49 (75.4)		172 (81.5)	
	Unknown or not reported	5 (7)		17 (23)		14 (21.5)		36 (17.1)	
BMI (kg/m^2^), mean (SD)			30.8 (8.34)		31.2 (7.50)		30.6 (6.63)		30.9 (7.56)
**Education level, n (%)**									
	High school or less^c^	24 (33.3)		28 (37.8)		24 (36.9)		76 (36)	
	Some college^d^	17 (23.6)		20 (27)		11 (16.9)		48 (22.6)	
	College or higher^e^	28 (38.9)		24 (32.4)		27 (41.5)		79 (37.4)	
	Unknown^f^	3 (4.2)		2 (2.7)		3 (4.6)		8 (3.8)	
**Employment status, n (%)**									
	Employed	7 (9.7)		9 (12.2)		10 (15.4)		26 (12.3)	
	Unemployed or retired	63 (87.5)		64 (86.5)		54 (83)		181 (85.8)	
	Unknown	2 (2.8)		1 (1.4)		1 (1.5)		4 (1.9)	
**Smoking status, n (%)**									
	Current smoker	14 (19.4)		19 (25.7)		9 (13.9)		42 (19.9)	
	Never smoked	30 (41.7)		18 (24.3)		28 (43.1)		76 (36)	
	Previously smoked	28 (38.9)		37 (50)		28 (43.1)		93 (44.1)	
**Opioid use, n (%)**									
	No	32 (44.4)		36 (48.7)		35 (53.9)		103 (48.8)	
	Yes	36 (50)		36 (48.7)		25 (38.5)		97 (46)	
	Not sure	4 (5.6)		2 (2.7)		5 (7.7)		11 (5.2)	
**Baseline recall pain severity, mean (SD)**									
	Worst pain	7.43 (1.71)		7.04 (1.76)		7.03 (1.53)		7.18 (1.67)	
	Average pain	6.00 (1.83)		6.14 (2.00)		6.03 (1.61)		6.07 (1.82)	
	Current pain	4.81 (2.73)		4.93 (2.71)		4.22 (2.85)		4.68 (2.77)	
**Baseline recall pain interference, mean (SD)**									
	Interfere with enjoyment of life	4.71 (3.05)		4.86 (3.05)		4.62 (2.94)		4.76 (3.00)	
	Interfere with daily general activities	5.35 (2.70)		5.47 (2.44)		4.74 (2.26)		5.22 (2.48)	

^a^T-APA: nontargeted auricular point acupressure.

^b^NT-APA: targeted auricular point acupressure.

^c^High school or less includes participants with no high school diploma or who graduated from high school or obtained a high school equivalency (General Educational Development).

^d^Some college includes those who attended an occupational, technical, or vocational program or completed some college without obtaining a degree.

^e^College or higher includes participants with an associate degree, bachelor’s degree, master’s degree, doctoral degree, or professional school degree.

^f^Unknown represents participants who did not report their education level.

A comparison of participants with versus without EMA data (Multimedia Appendix 2) revealed that those without EMA data were significantly older (mean age 72.15, SD 7.41 vs 69.36, SD 6.70 years; *P*=.01) and reported significantly lower pain interference with enjoyment of life at baseline (mean 3.80, SD 3.42) vs 4.74, SD 3.00; *P*=.04). No other baseline characteristics showed statistically significant between-groups differences.

EMA Compliance and Attrition

Based on the study design, EMA measurements were scheduled 3 times daily from baseline (day 0) through day 28 of the intervention period, for a total of 87 potential assessments over 29 days. The compliance analysis excluded 61 participants who did not respond to any EMA prompts. Among the remaining 211 participants, the overall compliance rate was 44.8%, and the attrition rate was 54%. Participant-level EMA compliance rates varied widely across individuals (Multimedia Appendix 3). There were no significant differences in compliance or attrition rates across the 3 treatment arms. Sex, race, analgesic use, opioid use, and baseline pain measurements were not significantly associated with compliance or attrition. However, age was significantly associated with compliance, with older participants exhibiting lower compliance rates (*F*_1,209_=6.96; *P*=.009), though age was not significantly related to attrition (*P*=.19).

### Patterns of EMA-Reported Pain Intensity and Pain Interference

**Figure 2:**
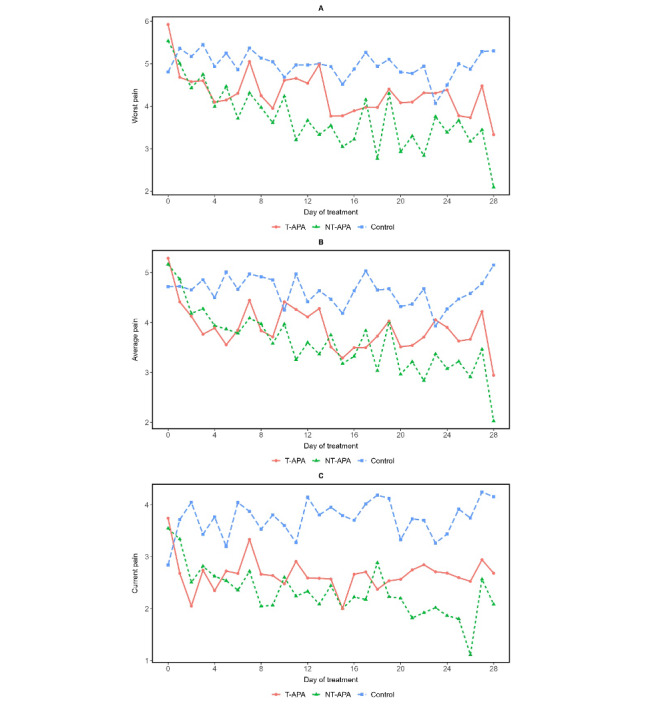
Patterns of ecological momentary assessment–reported pain intensity over the 4-week intervention period by treatment arm: worst pain (A), average pain (B), and current pain (C). NT-APA: nontargeted auricular point acupressure; T-APA: targeted auricular point acupressure.

Figure 2 shows the patterns of EMA-reported pain intensity, which were analyzed by examining the daily average scores of worst pain (Figure 2A), average pain (Figure 2B), and current pain (Figure 2C) over the 28-day intervention period across the 3 study arms, including T-APA, NT-APA, and education control. Both APA groups demonstrated a decreasing trend in worst and average pain over time, with consistently lower intensity levels compared to the control group. Although EMA-reported current pain scores showed some day-to-day variability, they remained generally lower in the APA groups throughout the intervention. These patterns suggest potential benefits of both APA interventions in reducing daily pain intensity.

**Figure 3:**
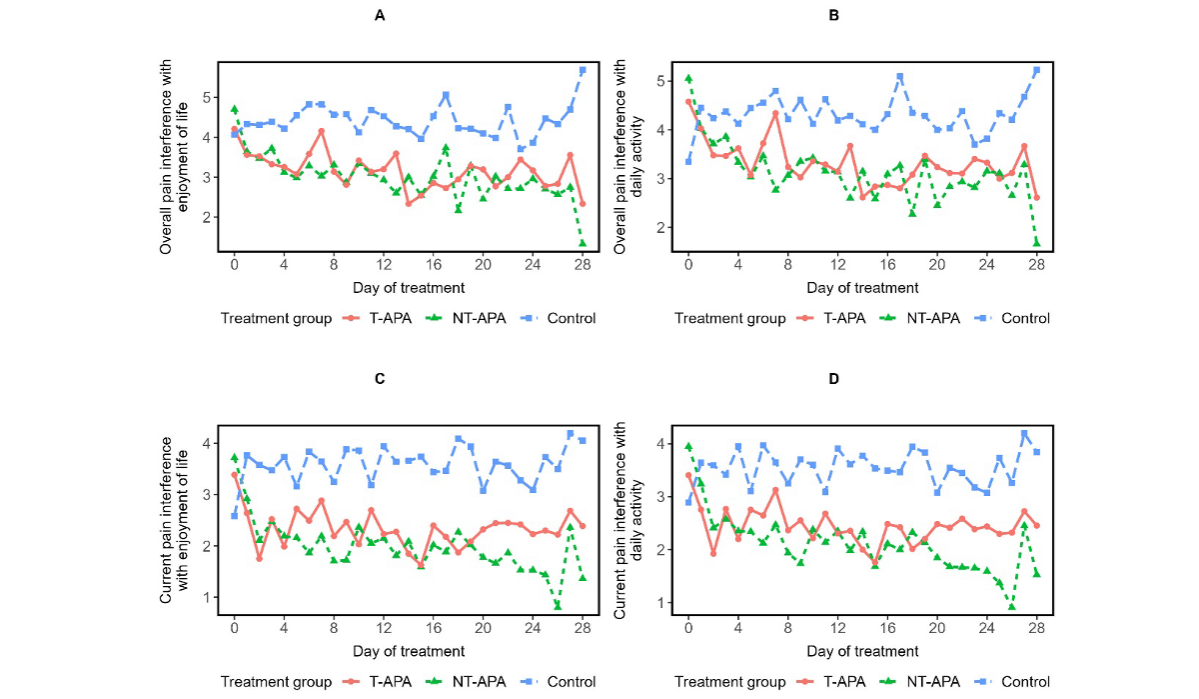
Patterns of ecological momentary assessment–reported overall pain interference with enjoyment of life (A) and daily activity (B), and current pain interference with enjoyment of life (C) and daily activity (D) over the 4-week auricular point acupressure by treatment arms. NT-APA: nontargeted auricular point acupressure; T-APA: targeted auricular point acupressure.

Figure 3 shows the EMA-reported overall pain interference. Daily average scores of overall pain interference with enjoyment in life (Figure 3A) and overall pain interference with daily activity (Figure 3B) over the 28-day period showed a decreasing trend in both the T-APA and NT-APA groups, with generally lower scores compared to the control group. Similar patterns were observed for EMA-reported current pain interference with enjoyment in life (Figure 3C) and current pain interference with daily activity (Figure 3D), suggesting that participants receiving APA experienced less disruption in daily functioning and enjoyment in life due to pain.

### Comparison of Pain Intensity and Pain Interference Outcomes Between EMA and 7-Day Recall

Weekly average EMA scores for pain intensity and interference were compared with corresponding weekly 7-day recall scores. Across all 4 weeks, EMA mean scores were consistently lower than recall-based ratings for worst and average pain (all *P*<.001). In contrast, EMA mean scores for interference with daily activity were significantly higher than recall scores across all weeks (all *P*≤.01). For interference with enjoyment of life, the recall data were collected only at Week 4, at which no significant difference was observed at that time point (*P*=.77; Table 2).

**Table 2:** Comparison of pain intensity and pain Interference between ecological momentary assessment (EMA) and 7-day recall using a 2-tailed paired *t* test.

Time		EMA, mean (SD)	7-day recalled, mean (SD)	*P* value
**Worst pain**				
	Week 1	4.81 (2.20)	6.45 (2.40)	<.001
	Week 2	4.44 (2.45)	6.24 (2.68)	<.001
	Week 3	4.21 (2.51)	6.23 (2.64)	<.001
	Week 4	4.09 (2.71)	5.81 (2.49)	<.001
**Average pain**				
	Week 1	4.39 (2.02)	4.91 (2.24)	<.001
	Week 2	4.17 (2.34)	4.66 (2.41)	<.001
	Week 3	3.89 (2.44)	4.65 (2.48)	<.001
	Week 4	3.75 (2.60)	4.33 (2.39)	<.001
**Overall pain interference with daily activity**				
	Week 1	3.93 (2.45)	2.82 (1.04)	<.001
	Week 2	3.63 (2.62)	2.76 (1.04)	<.001
	Week 3	3.44 (2.65)	2.84 (1.07)	.002
	Week 4	3.45 (2.77)	2.86 (1.06)	.01
**Overall pain interference with enjoyment of life**				
	Week 1	3.86 (2.45)	—^a^	—
	Week 2	3.59 (2.72)	—	—
	Week 3	3.39 (2.76)	—	—
	Week 4	3.40 (2.84)	3.58 (3.18)	.77

^a^Not applicable.

Across the intervention period, EMA and 7-day recall ratings were highly correlated for both pain intensity and pain interference outcomes. Spearman correlation coefficients demonstrated strong associations between EMA and 7-day recall ratings across all 4 weeks of the intervention. For both worst and average pain, correlations between EMA and recall scores remained consistently high (ranging from 0.73 to 0.95, all *P*<.001; Table 3). Similarly, overall pain interference with daily activity showed moderate to strong correlations between EMA and recall across the weeks (ranging from 0.66 to 0.80, all *P*<.001). In Week 4, when recall data for overall pain interference with enjoyment of life were available, correlations with EMA were also moderate (=0.59 for recall and 0.56 for EMA, both *P*<.001; Table 3).

**Table 3:** Spearman correlation coefficients of pain intensity and pain interference between ecological momentary assessment (EMA) and 7-day recall for every week throughout the intervention period.

Pain measures and week		Worst pain			Average pain			Overall pain interference with daily activity			Overall pain interference with enjoyment of life	
		Recall	EMA	Recall		EMA	Recall		EMA	Recall		EMA
**Week 1: worst pain**												
	Recall	—^a^	0.61	0.74^b^		0.62^b^	0.53^b^		0.56^b^	—		—
	EMA	0.61^b^	—	0.73^b^		0.90^b^	0.56^b^		0.83^b^	—		—
**Week 2: worst pain**												
	Recall	—	0.80^b^	0.90^b^		0.76^b^	0.71^b^		0.79^b^	—		—
	EMA	0.80^b^	—	0.86^b^		0.92^b^	0.70^b^		0.89^b^	—		—
**Week 3: worst pain**												
	Recall	—	0.75^b^	0.80^b^		0.72^b^	0.53^b^		0.71^b^	—		—
	EMA	0.75^b^	—	0.85^b^		0.92^b^	0.60^b^		0.88^b^	—		—
**Week 4: worst pain**												
	Recall	—	0.74^b^	0.79^b^		0.74^b^	0.62^b^		0.70^b^	0.58^b^		0.68^b^
	EMA	0.74^b^	—	0.78^b^		0.95^b^	0.62^b^		0.89^b^	0.53^b^		0.87^b^
**Week 1: average pain**												
	Recall	0.74^b^	0.62^b^	—		0.78^b^	0.56^b^		0.73^b^	—		—
	EMA	0.73^b^	0.90^b^	0.78^b^		—	0.56^b^		0.89^b^	—		—
**Week 2: average pain**												
	Recall	0.90^b^	0.86^b^	—		0.86^b^	0.74^b^		0.86^b^	—		—
	EMA	0.76^b^	0.92^b^	0.86^b^		—	0.67^b^		0.78^b^	—		—
**Week 3: average pain**												
	Recall	0.80^b^	0.85^b^	—		0.84^b^	0.66^b^		0.84^b^	—		—
	EMA	0.72^b^	0.92^b^	0.84^b^		—	0.64^b^		0.93^b^	—		—
**Week 4: average pain**												
	Recall	0.79^b^	0.78^b^	—		0.82^b^	0.69^b^		0.81^b^	0.59^b^		0.79^b^
	EMA	0.74^b^	0.95^b^	0.78^b^		—	0.62^b^		0.94^b^	0.56^b^		0.92^b^
**Week 1: overall pain interference with daily activity**												
	Recall	0.53^b^	0.56^b^	0.56^b^		0.56^b^	—		0.66^b^	—		—
	EMA	0.56^b^	0.83^b^	0.83^b^		0.73^b^	0.66^b^		—	—		—
**Week 2: overall pain interference with daily activity**												
	Recall	0.71^b^	0.70^b^	0.74^b^		0.67^b^	—		0.78^b^	—		—
	EMA	0.79^b^	0.89^b^	0.86^b^		0.91^b^	0.78^b^		—	—		—
**Week 3: overall pain interference with daily activity**												
	Recall	0.53^b^	0.60^b^	0.66^b^		0.64^b^	—		0.80^b^	—		—
	EMA	0.71^b^	0.88^b^	0.84^b^		0.93^b^	0.70^b^		—	—		—
**Week 4: overall pain interference with daily activity**												
	Recall	0.62^b^	0.62^b^	0.69^b^		0.62^b^	—		0.72^b^	0.64^b^		0.69^b^
	EMA	0.70^b^	0.89^b^	0.81^b^		0.94^b^	0.72^b^		—	0.62^b^		0.95^b^

^a^Not applicable.

^b^*P*<.001.

### The Effects of APA Practice and Medication Use on EMA-Reported Pain Intensity

Both T-APA and NT-APA interventions significantly reduced EMA-reported pain intensity across 3 specific variables, including worst pain, average pain, and current pain. As shown in Table 4, participants in the T-APA group experienced significant reductions in EMA-reported worst pain (β=–0.98, SE 0.33; *P*<.001), EMA-reported average pain (β=–0.93, SE 0.30; *P*<.001), and EMA-reported current pain (β=–1.01, SE 0.36; *P*=.006; Table 4). Similarly, the NT-APA group showed significant reductions in EMA-reported worst pain (β=–0.74, SE 0.12; *P*<.001), EMA-reported average pain (β=–1.02, SE 0.30; *P*=.001), and EMA-reported current pain (β=–1.26, SE 0.37; *P*=.001) compared to the control group. When comparing T-APA and NT-APA directly, there were no statistically significant differences in any of the momentary pain outcomes, suggesting comparable efficacy between the 2 APA protocols.

Adherence to APA practice was variably associated with EMA pain outcomes. Longer pressing time per session (more than 3 minutes) was significantly associated with reduced EMA-reported current pain (β=–0.28, SE 0.13; *P*=.02). In contrast, pressing more than 3 times per day was unexpectedly linked with increased EMA-reported worst pain (β=0.29, SE 0.15; *P*=.04) and EMA-reported average pain (β=0.32, SE 0.13; *P*=.01). Total daily pressing time greater than 9 minutes was not significantly associated with changes in any EMA-reported pain variable, indicating that pressing duration per instance may be more relevant than cumulative daily time.

Baseline pain characteristics, measured by a 7-day recall self-report questionnaire, also predicted EMA-reported pain outcomes. Use of analgesic medications was associated with increased EMA-reported pain across all domains, including worst pain (β=0.74, SE 0.12; *P*<.001), average pain (β=0.47, SE 0.11; *P*<.001), and current pain (β=0.74, SE 0.14; *P*<.001). Higher baseline recall of worst pain was associated with greater levels of all EMA-reported pain outcomes, such as worst pain (β=0.51, SE 0.09; *P*<.001), average pain (β=0.31, SE 0.10, *P*=.003), and current pain (β=0.27, SE 0.11; *P*=.02). Similarly, higher baseline recall of pain interference with daily activities was associated with increased EMA-reported worst pain (β=0.20, SE 0.06, *P*=.001) and EMA-reported average pain (β=0.19, SE 0.06, *P*=.001). Furthermore, baseline recall, average pain, and current pain were significant predictors of their corresponding EMA-reported pain outcomes.

Among demographic variables, age was the only significant factor associated with EMA-reported pain outcomes. Older age was linked with reduced EMA-reported worst pain (β=–0.07, SE 0.02; *P*=.001) and EMA-reported average pain (β=–0.06, SE 0.02; *P*=.001), though it was not significantly related to EMA-reported current pain. Other demographic and clinical characteristics—including sex, race, employment status, smoking status, opioid use, and baseline pain interference with enjoyment of life—were not significantly associated with any EMA-reported pain outcomes.

**Table 4:** The estimated coefficients and SEs of the main effects on EMA-reported pain intensity.

EMA-reported pain intensity			Worst pain					Average pain					Current pain		
			Estimate (SE)		*P* value		Estimate (SE)			*P* value		Estimate (SE)			*P* value
**Group (control as a comparison group)**															
	T-APA^a^	–0.98 (0.33)		<.001^b^		–0.93 (0.30)			<.001^b^		–1.01 (0.36)			.006^b^	
	NT-APA^c^	–0.74 (0.12)		<.001^b^		–1.02 (0.30)			.001^b^		–1.26 (0.37)			.001^b^	
	Control	Ref		Ref		Ref			Ref		Ref			Ref	
**Group (NT-APA as a comparison group)**															
	T-APA	0.28 (0.29)		.34		0.09 (0.27)			.74		0.25 (0.34)			.47	
	NT-APA	Ref		Ref		Ref			Ref		Ref			Ref	
	Control	1.25 (0.34)		<.001^b^		1.02 (0.30)			<.001^b^		1.26 (0.37)			<.001^b^	
**Pressing frequency (times/day)**															
	≥3	0.29 (0.15)		.04^b^		0.32 (0.13)			.01^b^		—^d^			—	
	<3	Ref		Ref		Ref			Ref		—			—	
**Minutes per pressing**															
	≥3	–0.25 (0.17)		.14		–0.22 (0.15)			.12		–0.28 (0.13)			.02^b^	
	<3	Ref		Ref		Ref			Ref		Ref			Ref	
**Total pressing time (minutes)**															
	≥9	—		—		—			—		—			—	
	<9	—		—		—			—		—			—	
**Analgesic use (confounding)**															
	Yes	0.74 (0.12)		<.001^b^		0.47 (0.11)			<.001^b^		0.74 (0.14)			<.001^b^	
	No	Ref		Ref		Ref			Ref		Ref			Ref	
	Day of treatment	–0.04 (0.00)		<.001^b^		–0.03 (0.00)			<.001^b^		–0.01 (0.00)			.001^b^	
**Covariates** ^e^															
	Age	–0.07 (0.02)		.001^b^		–0.06 (0.02)			.001^b^		—			—	
	Recall worst pain	0.51 (0.09)		<.001^b^		0.31 (0.10)			.003^b^		0.27 (0.11)			.02^b^	
	Recall average pain	—		—		0.27 (0.09)			.002^b^		—			—	
	Recall current pain	—		—		—			—		0.36 (0.07)			<.001^b^	
	Recall pain interference with daily activities	0.20 (0.06)		.001^b^		0.19 (0.06)			.001^b^		—			—	

^a^T-APA: targeted auricular point acupressure.

^b^*P*<.05.

^c^NT-APA: nontargeted auricular point acupressure.

^d^Variable not selected for the final model due to lack of statistical significance or contribution to model fit.

^e^Models adjusted for baseline demographics (age, sex, race, employment, smoking, and opioid use) and baseline recall pain variables (intensity and interference).

### The Effects of APA Practice and Medication Use on EMA-Reported Pain Interference

Linear mixed-effects models showed that both APA interventions were significantly associated with reductions in EMA-reported pain interference outcomes compared to the control group. T-APA significantly reduced EMA-reported overall pain interference with enjoyment of life (β=–1.72, SE 0.37; *P*<.001), overall pain interference with daily activity (β=–1.41, SE 0.34; *P*=.001), current pain interference with enjoyment of life (β=–1.28, SE 0.40; *P*=.003), and current pain interference with daily activity (β=–1.27, SE 0.37; *P*=.001). NT-APA produced similar reductions in all EMA-reported pain interference outcomes, with effect sizes ranging from β=–1.21 to –1.50 (all *P*≤.002; Table 5).

**Table 5:** The estimated coefficients and SEs of the main effects on pain interference.

			EMA^a^-reported overall pain interference										EMA-reported current pain interference							
			Enjoyment of life					Daily activity					Enjoyment of life					Daily activity		
			Estimate (SE)		*P* value		Estimate (SE)			*P* value		Estimate (SE)			*P* value		Estimate (SE)			*P* value
**Group**																				
	T-APA^b^	–1.72 (0.37)		<.001^c^		–1.41 (0.34)			.001^c^		–1.28 (0.40)			.003^c^		–1.27 (0.37)			.001^c^	
	NT-APA^d^	–1.21 (0.37)		.002^c^		–1.10 (0.34)			.002^c^		–1.45 (0.41)			<.001^c^		–1.50 (0.38)			<.001^c^	
	Control	Ref		Ref		Ref			Ref		Ref			Ref		Ref			Ref	
**Pressing frequency (times/day)**																				
	≥3	0.44 (0.14)		.002^c^		—^e^			—		—			—		—			—	
	<3	Ref		Ref		—			—		—			—		—			—	
**Minutes per pressing**																				
	≥3	–0.28 (0.14)		.04^c^		—			—		–0.21 (0.10)			.03^c^		–0.20 (0.10)			.047^c^	
	<3	Ref		Ref		—			—		Ref			Ref		Ref			Ref	
**Total pressing time (minutes)**																				
	≥9	—		—		0.33 (0.13)			.009^c^		—			—		—			—	
	<9	—		—		Ref			Ref		—			—		—			—	
**Analgesic use**																				
	Yes	0.68 (0.14)		<.001^c^		0.76 (0.14)			<.001^c^		0.58 (0.14)			<.001^c^		0.65 (0.13)			<.001^c^	
	No	Ref		Ref		Ref			Ref		Ref			Ref		Ref			Ref	
	Day of treatment	–0.02 (0.00)		<.001^c^		–0.02 (0.00)			<.001^c^		–0.02 (0.00)			<.001^c^		–0.02 (0.00)			<.001^c^	
**Covariates** ^f^																				
	Age	—		—		—			—		—			—		—			—	
	Recall worst pain	0.53 (0.10)		<.001^c^		0.46 (0.10)			<.001^c^		—			—		—			—	
	Recall average pain	—		—		—			—		—			—		—			—	
	Recall current pain	—		—		—			—		0.34 (0.07)			<.001^c^		0.33 (0.06)			<.001* ^c^	
	Recall pain interference with enjoyment of life	0.35 (0.07)		<.001^c^		—			—		0.23 (0.06)			<.001^c^		—			—	
	Recall pain interference with daily activities	—		—		0.27 (0.05)			<.001^c^		—			—		0.30 (0.07)			<.001^c^	

^a^EMA: ecological momentary assessment.

^b^T-APA: targeted auricular point acupressure.

^c^*P*<.05.

^d^NT-APA: nontargeted auricular point acupressure.

^e^Variable not selected.

^f^Models adjusted for baseline demographics (age, sex, race, employment, smoking, and opioid use) and baseline recall pain variables (intensity and interference).

APA practice behaviors were variably associated with EMA-reported pain interference outcomes. Longer pressing duration (more than 3 minutes per session) was significantly associated with lower EMA-reported pain interference in several domains. Participants with longer pressing time reported reduced EMA-reported overall pain interference with enjoyment of life (β=–0.28, SE 0.17; *P*=.04), current pain interference with enjoyment of life (β=—0.21, SE 0.10; *P*=.03), and current pain interference with daily activity (β=—0.20, SE 0.10; *P*=.047), but not with overall pain interference with daily activity. However, pressing more than 3 times daily was associated with greater EMA-reported overall pain interference with enjoyment of life (β=0.44, SE 0.14; *P*=.002), and total pressing time greater than 9 minutes per day was linked to greater EMA-reported overall pain interference with daily activity (β=0.33, SE 0.13; *P*=.009; Table 5).

Analgesic use was consistently associated with increased EMA-reported pain interference across all outcomes (β=0.58-0.76, all *P*<.001). Higher baseline recall pain interference scores predicted greater EMA-reported interference during the intervention. Specifically, baseline recall pain interference with enjoyment of life was associated with greater EMA-reported overall (β=0.35, SE 0.07; *P*<.001) and current (β=0.23, SE 0.06; *P*<.001) pain interference with enjoyment in life. Similarly, baseline recall pain interference with daily activities predicted greater EMA-reported overall (β=0.27, SE 0.05; *P*<.001) and current (β=0.30, SE 0.07; *P*<.001) pain interference with daily activity (Table 5).

Baseline recall of pain intensity was significantly associated with EMA-reported pain interference outcomes. Higher baseline recall of worst pain was associated with greater EMA-reported overall pain interference with enjoyment of life (β=0.53, SE 0.10; *P*<.001) and daily activity (β=0.46, SE 0.10; *P*<.001). Higher baseline recall of current pain was significantly associated with both EMA-reported current pain interference with enjoyment of life and daily activity (β=0.33-0.34, both *P*<.001). No significant associations were observed for age, sex, race, smoking status, employment, baseline opioid use, or baseline recall of average pain (Table 5).

## Discussion

### Principal Findings

This secondary analysis of EMA data from an RCT supports the real-time effectiveness of APA in managing cLBP among older adults. Our results build on the original trial’s finding [6], demonstrating sustained improvements in self-reported pain and function while uncovering the dynamic relationship between APA practice and day-to-day symptom trajectories.

A key contribution of this study is the use of EMA to capture real-time symptom fluctuations. Compared to traditional 7-day recall measures, EMA consistently recorded lower pain intensity but higher pain interference scores. These findings align with prior research suggesting that retrospective methods may overestimate pain severity due to memory distortion and underestimate real-time disruptions to daily life [10,12,23,24]. This discrepancy may reflect the tendency of individuals to experience more interference in the moment but recall less of it afterward [25]. However, measuring interference with EMA remains methodologically complex; a prior study has found that interference ratings may be more variable and less reliable than intensity ratings in real-time formats [26]. Further research should evaluate the psychometric stability of EMA-based interference items to inform appropriate use in future trials.

This study was built on previous research by Yeh et al [27] and Lin et al [12], but with a larger sample size. EMA compliance in this study was lower (44.8%) compared to 87% in previous reports [12], and the attrition rate was higher (54% vs 17%-32%) [27]. Age was a significant predictor of lower compliance, suggesting potential barriers to EMA participation among older adults. While previous meta-analyses have found that younger participants may show quicker declines in EMA adherence over time [28], older adults may face distinct challenges, such as fatigue, cognitive burden, or technological limitations. A study recommends limiting EMA to 1 week to reduce the burden [29]; such an approach may hinder the ability to observe longitudinal treatment effects. An alternative strategy involves EMA burst designs, which collect intensive real-time data over brief intervals (eg, several days), repeated periodically over the course of an intervention [30,31]. This design may offer a compromise between detailed data tracking and participant burden, particularly for older adults or those with limited digital literacy.

The study also examined APA practice behaviors and their associations with pain outcomes. Pressing for more than 3 minutes per session was associated with reduced EMA-reported pain outcomes, including lower current pain intensity and reduced interference across several domains, consistent with prior APA dosing recommendations [27]. These findings support the importance of adequate per-session stimulation time. In contrast, more frequent pressing (≥3 times/day) was associated with higher EMA-reported worst and average pain intensity, as well as greater overall interference with enjoyment of life. Additionally, total daily pressing time over 9 minutes was linked to greater interference with daily activity, though it was not associated with pain intensity. These associations may reflect reverse causality, where individuals experiencing more severe symptoms press more frequently or for longer durations to manage discomfort. Prior studies have suggested that greater APA frequency improves recall outcomes [12,27], but this discrepancy highlights the importance of real-time assessment and individualized dosing. Taken together, these results suggest that per-session duration may be more influential than cumulative daily time and that optimal APA dosing strategies may vary across individuals and symptom profiles.

Exploratory analyses of baseline characteristics revealed that higher baseline recall worst, and average pain levels, which were associated with higher EMA-reported pain intensity and interference during the intervention, consistent with patterns observed in systematic reviews of cLBP populations [32]. Analgesic use was also associated with increased symptom burden, possibly reflecting higher baseline severity and treatment-seeking behaviors. Interestingly, older age was associated with lower EMA-reported pain intensity, suggesting potential differences in pain perception or response to APA in older individuals. These results underscore the importance of personalized APA strategies that take into account symptom severity, medication use, and age.

While these findings support the promise of dynamic, personalized interventions, real-world apps will require adjustments to the delivery infrastructure. In the RCT, participants received automated reminders, technical support, and weekly reinforcement—all of which would be difficult to replicate in standard care. Participants also used uniform smartphones and a simplified app. In practice, device variability, digital literacy, and lack of support may hinder EMA engagement [33]. Prior studies have shown that technological burden, lack of training, and inconsistent access to compatible devices are common barriers to sustained EMA use in older adult and chronic pain populations [34,35]. Future adaptations should prioritize user-friendly design, embedded prompts, automated troubleshooting, and brief onboarding to support participation across diverse settings.

Finally, the integration of EMA into APA trials opens the door to future development of just-in-time adaptive approaches that dynamically tailor support based on real- time symptom data. By detecting periods of elevated pain or interference in real time, these systems could prompt users to engage in APA or other self-management strategies at moments of greatest need. While not tested in this study, our findings support the feasibility of capturing real-time pain dynamics—a critical step toward implementing adaptive, personalized digital interventions [36-38]. Incorporating the features of just-in-time adaptive interventions into mobile platforms may further optimize APA delivery by enabling real-time behavioral support, especially for populations managing fluctuating symptoms.

### Limitations

This study has several limitations that should be acknowledged. First, as a secondary analysis of an RCT, the inclusion criterion of having at least one EMA entry led to the exclusion of 61 participants, who were significantly older and had lower baseline pain interference scores. This introduces potential selection bias and limits generalizability to older adults with lower digital literacy or more advanced physical or cognitive limitations. Second, despite promising findings in a prior pilot study, EMA compliance in this trial was modest (44.8%), and attrition was high (54%), particularly among older participants. This may have affected the completeness and representativeness of real-time data and reduced statistical power to detect more nuanced effects. The high frequency of EMA prompts (up to 3 times daily for 4 weeks) may have contributed to measurement fatigue and burden, which are known challenges in older adult populations using mobile health tools [33-35]. Moreover, while the use of EMA allowed for rich, real-time data, the act of frequent self-monitoring may itself influence behavior or symptom reporting, a phenomenon known as EMA reactivity, which was not assessed in this study [39].

Additionally, pressing behaviors were self-reported and not objectively verified, which may introduce recall or social desirability bias, particularly in reporting APA adherence. The study’s relatively short intervention duration (4 weeks) also limited the ability to assess long-term outcomes or sustained benefits. Although the use of standardized smartphones and technical support ensured uniformity during the trial, these supports may not be available in real-world practice, where diverse device use, technical variability, and digital inequity may pose barriers to implementation [30,31,38]. Finally, both T-APA and NT-APA protocols showed similar effects, which may limit the interpretation of treatment specificity and suggest the need for further mechanistic research to understand the role of point selection.

### Conclusions

This study provides evidence that APA effectively reduces pain intensity and improves daily functioning and quality of life in older adults with cLBP. The use of EMA offers a valuable method for capturing real-time pain experiences, mitigating recall bias, and providing a more accurate assessment of treatment effects. However, challenges related to EMA compliance highlight the need for refined intervention protocols. Future research should focus on optimizing APA regimens, extending intervention duration, and improving EMA adherence to enhance its applicability in clinical practice. By addressing these considerations, APA has the potential to serve as a widely accessible and effective self-management strategy for chronic pain relief.

## Data Availability

The datasets analyzed will be available from the corresponding author on request.

## Appendix

Multimedia Appendix 1 CONSORT-eHEALTH checklist (V 1.6.1).

Multimedia Appendix 2 Demographics and participants' characteristics.

Multimedia Appendix 3 Distribution of individual EMA compliance rates among participants.

## Abbreviations

APA: auricular point acupressure
BPI-SF: Brief Pain Inventory–Short Form
cLBP: chronic low back pain
CONSORT-EHEALTH: Consolidated Standards of Reporting Trials of Electronic and Mobile Health Applications and Online Telehealth
EMA: ecological momentary assessment
NT-APA: nontargeted auricular point acupressure
RCT: randomized controlled trial
T-APA: targeted auricular point acupressure
