# Supplementary material for: Smartphone-Based Ecological Momentary Assessment of Pain in Older Adults Undergoing Auricular Point Acupressure for Chronic Low Back Pain: Secondary Analysis of a Randomized Controlled Trial
Source: JMIR Form Res. 2026 Mar 4;10:e79612. doi: 10.2196/79612 (PMC13000383; doi:10.2196/79612)
Supplement: Multimedia Appendix 1 [file formative_v10i1e79612_app1.pdf]

CONSORT-EHEALTH (V 1.6.1) - Submission/Publication Form

The CONSORT-EHEALTH checklist is intended for authors of randomized trials evaluating web-based and Internet-based applications/interventions, including mobile interventions, electronic games (incl multiplayer games), social media, certain telehealth applications, and other interactive and/or networked electronic applications. Some of the items (e.g. all subitems under item 5 - description of the intervention) may also be applicable for other study designs.

The goal of the CONSORT EHEALTH checklist and guideline is to be
a) a guide for reporting for authors of RCTs,
b) to form a basis for appraisal of an ehealth trial (in terms of validity)

CONSORT-EHEALTH items/subitems are MANDATORY reporting items for studies published in the Journal of Medical Internet Research and other journals / scientific societies endorsing the checklist.

Items numbered 1., 2., 3., 4a., 4b etc are original CONSORT or CONSORT-NPT (non-pharmacologic treatment) items.
Items with Roman numerals (i., ii, iii, iv etc.) are CONSORT-EHEALTH extensions/clarifications.

As the CONSORT-EHEALTH checklist is still considered in a formative stage, we would ask that you also RATE ON A SCALE OF 1-5 how important/useful you feel each item is FOR THE PURPOSE OF THE CHECKLIST and reporting guideline (optional).

Mandatory reporting items are marked with a red *.
In the textboxes, either copy & paste the relevant sections from your manuscript into this form - please include any quotes from your manuscript in QUOTATION MARKS,
or answer directly by providing additional information not in the manuscript, or elaborating on why the item was not relevant for this study.

YOUR ANSWERS WILL BE PUBLISHED AS A SUPPLEMENTARY FILE TO YOUR PUBLICATION IN JMIR AND ARE CONSIDERED PART OF YOUR PUBLICATION (IF ACCEPTED).
Please fill in these questions diligently. Information will not be copyedited, so please use proper spelling and grammar, use correct capitalization, and avoid abbreviations.

DO NOT FORGET TO SAVE AS PDF _AND_ CLICK THE SUBMIT BUTTON SO YOUR ANSWERS ARE IN OUR DATABASE !!!

Citation Suggestion (if you append the pdf as Appendix we suggest to cite this paper in the caption):
Eysenbach G, CONSORT-EHEALTH Group
CONSORT-EHEALTH: Improving and Standardizing Evaluation Reports of Web-based and Mobile Health Interventions
J Med Internet Res 2011;13(4):e126

URL: http://www.jmir.org/2011/4/e126/
doi: 10.2196/jmir.1923
PMID: 22209829

chitchanok.ben@gmail.com Switch account

Draft not saved

Not shared

* Indicates required question

Your name *

First Last

Chitchanok Benjasirisan

Primary Affiliation (short), City, Country *

University of Toronto, Toronto, Canada

Johns Hopkins School of Nursing, Baltimore, M

Your e-mail address *

abc@gmail.com

cbenjas1@jh.edu

Title of your manuscript *

Provide the (draft) title of your manuscript.

Smartphone-based Ecological Momentary Assessment of Pain During Auricular Point Acupressure in Older Adults with Chronic Low Back Pain: a Randomized Controlled Trial

Name of your App/Software/Intervention *

If there is a short and a long/alternate name, write the short name first and add the long name in brackets.

EarAPA

Evaluated Version (if any)

e.g. "V1", "Release 2017-03-01", "Version 2.0.27913"

Your answer

Language(s) *

What language is the intervention/app in? If multiple languages are available, separate by comma (e.g. "English, French")

English

URL of your Intervention Website or App

e.g. a direct link to the mobile app on app in appstore (itunes, Google Play), or URL of the website. If the intervention is a DVD or hardware, you can also link to an Amazon page.

https://play.google.com/store/apps/details?id=technology.chi.earapa&hl=en

URL of an image/screenshot (optional)

Your answer

Accessibility *

Can an enduser access the intervention presently?

- ○ access is free and open
- ○ access only for special usergroups, not open
- ○ access is open to everyone, but requires payment/subscription/in-app purchases
- ○ app/intervention no longer accessible
- ◉ Other: The download is free but requires an access key from the researcher.

Primary Medical Indication/Disease/Condition *

e.g. "Stress", "Diabetes", or define the target group in brackets after the condition, e.g. "Autism (Parents of children with)", "Alzheimers (Informal Caregivers of)"

Older adults with chronic low back pain

Primary Outcomes measured in trial *

comma-separated list of primary outcomes reported in the trial

Pain intensity and pain interference

Secondary/other outcomes

Are there any other outcomes the intervention is expected to affect?

7-day recall data, captured weekly ratings of worst pain, average pain, overall pain interference with daily activities, and overall pain interference with enjoyment of life, analgesic use, and Auricular Point Acupressure (APA) practice evaluated based on three factors: the frequency of pressing the taped seeds, minutes per pressing, and total pressing time.

Recommended "Dose" *

What do the instructions for users say on how often the app should be used?

- ( ) Approximately Daily
- ( ) Approximately Weekly
- ( ) Approximately Monthly
- ( ) Approximately Yearly
- ( ) "as needed"
- (•) Other: 3 times per day

Approx. Percentage of Users (starters) still using the app as recommended after 3 months *

- (•) unknown / not evaluated
- ( ) 0-10%
- ( ) 11-20%
- ( ) 21-30%
- ( ) 31-40%
- ( ) 41-50%
- ( ) 51-60%
- ( ) 61-70%
- ( ) 71%-80%
- ( ) 81-90%
- ( ) 91-100%
- ( ) Other:

Overall, was the app/intervention effective? *

- (selected) yes: all primary outcomes were significantly better in intervention group vs control
- partly: SOME primary outcomes were significantly better in intervention group vs control
- no statistically significant difference between control and intervention
- potentially harmful: control was significantly better than intervention in one or more outcomes
- inconclusive: more research is needed
- Other:

Article Preparation Status/Stage *

At which stage in your article preparation are you currently (at the time you fill in this form)

- not submitted yet - in early draft status
- (selected) not submitted yet - in late draft status, just before submission
- submitted to a journal but not reviewed yet
- submitted to a journal and after receiving initial reviewer comments
- submitted to a journal and accepted, but not published yet
- published
- Other:

Journal *

If you already know where you will submit this paper (or if it is already submitted), please provide the journal name (if it is not JMIR, provide the journal name under "other")

- ○ not submitted yet / unclear where I will submit this
- ○ Journal of Medical Internet Research (JMIR)
- ○ JMIR mHealth and UHealth
- ○ JMIR Serious Games
- ○ JMIR Mental Health
- ○ JMIR Public Health
- ◉ JMIR Formative Research
- ○ Other JMIR sister journal
- ○ Other:

Is this a full powered effectiveness trial or a pilot/feasibility trial? *

- ○ Pilot/feasibility
- ◉ Fully powered

Manuscript tracking number *

If this is a JMIR submission, please provide the manuscript tracking number under "other" (The ms tracking number can be found in the submission acknowledgement email, or when you login as author in JMIR. If the paper is already published in JMIR, then the ms tracking number is the four-digit number at the end of the DOI, to be found at the bottom of each published article in JMIR)

- ◉ no ms number (yet) / not (yet) submitted to / published in JMIR
- ○ Other:

TITLE AND ABSTRACT

1a) TITLE: Identification as a randomized trial in the title

1a) Does your paper address CONSORT item 1a? *

I.e does the title contain the phrase "Randomized Controlled Trial"? (if not, explain the reason under "other")

- (●) yes
- ( ) Other:

1a-i) Identify the mode of delivery in the title

Identify the mode of delivery. Preferably use “web-based” and/or “mobile” and/or “electronic game” in the title. Avoid ambiguous terms like “online”, “virtual”, “interactive”. Use “Internet-based” only if Intervention includes non-web-based Internet components (e.g. email), use “computer-based” or “electronic” only if offline products are used. Use “virtual” only in the context of “virtual reality” (3-D worlds). Use “online” only in the context of “online support groups”. Complement or substitute product names with broader terms for the class of products (such as “mobile” or “smart phone” instead of “iphone”), especially if the application runs on different platforms.

	1	2	3	4	5	
subitem not at all important	( )	( )	( )	( )	(●)	essential

Clear selection

Does your paper address subitem 1a-i? *

Copy and paste relevant sections from manuscript title (include quotes in quotation marks "like this" to indicate direct quotes from your manuscript), or elaborate on this item by providing additional information not in the ms, or briefly explain why the item is not applicable/relevant for your study

"Smartphone-based Ecological Momentary Assessment of Pain During Auricular Point Acupressure in Older Adults with Chronic Low Back Pain: a Randomized Controlled Trial"

1a-ii) Non-web-based components or important co-interventions in title

Mention non-web-based components or important co-interventions in title, if any (e.g., “with telephone support”).

	1	2	3	4	5	
subitem not at all important	○	○	○	○	◉	essential

Clear selection

Does your paper address subitem 1a-ii?

Copy and paste relevant sections from manuscript title (include quotes in quotation marks "like this" to indicate direct quotes from your manuscript), or elaborate on this item by providing additional information not in the ms, or briefly explain why the item is not applicable/relevant for your study

"Auricular Point Acupressure"

1a-iii) Primary condition or target group in the title

Mention primary condition or target group in the title, if any (e.g., “for children with Type I Diabetes”) Example: A Web-based and Mobile Intervention with Telephone Support for Children with Type I Diabetes: Randomized Controlled Trial

	1	2	3	4	5	
subitem not at all important	○	○	○	○	◉	essential

Clear selection

Does your paper address subitem 1a-iii? *

Copy and paste relevant sections from manuscript title (include quotes in quotation marks "like this" to indicate direct quotes from your manuscript), or elaborate on this item by providing additional information not in the ms, or briefly explain why the item is not applicable/relevant for your study

"in Older Adults with Chronic Low Back Pain"

1b) ABSTRACT: Structured summary of trial design, methods, results, and conclusions

NPT extension: Description of experimental treatment, comparator, care providers, centers, and blinding status.

1b-i) Key features/functionalities/components of the intervention and comparator in the METHODS section of the ABSTRACT

Mention key features/functionalities/components of the intervention and comparator in the abstract. If possible, also mention theories and principles used for designing the site. Keep in mind the needs of systematic reviewers and indexers by including important synonyms. (Note: Only report in the abstract what the main paper is reporting. If this information is missing from the main body of text, consider adding it)

	1	2	3	4	5	
subitem not at all important	○	○	○	○	◉	essential

Clear selection

Does your paper address subitem 1b-i? *

Copy and paste relevant sections from the manuscript abstract (include quotes in quotation marks "like this" to indicate direct quotes from your manuscript), or elaborate on this item by providing additional information not in the ms, or briefly explain why the item is not applicable/relevant for your study

"This study evaluates the effectiveness of self-administered APA, applied at least three times daily for three minutes per session, using smartphone-based Ecological Momentary Assessment (EMA) to capture real-time pain outcomes."

"A three-arm randomized controlled trial included 272 adults aged ≥60 years with cLBP, randomized into targeted APA (T-APA, n=92), non-targeted APA (NT-APA, n=91), and education control (n=89) groups."

1b-ii) Level of human involvement in the METHODS section of the ABSTRACT

Clarify the level of human involvement in the abstract, e.g., use phrases like “fully automated” vs. “therapist/nurse/care provider/physician-assisted” (mention number and expertise of providers involved, if any). (Note: Only report in the abstract what the main paper is reporting. If this information is missing from the main body of text, consider adding it)

	1	2	3	4	5	
subitem not at all important	○	○	◉	○	○	essential

Clear selection

Does your paper address subitem 1b-ii?

Copy and paste relevant sections from the manuscript abstract (include quotes in quotation marks "like this" to indicate direct quotes from your manuscript), or elaborate on this item by providing additional information not in the ms, or briefly explain why the item is not applicable/relevant for your study

"This study evaluates the effectiveness of self-administered APA, applied at least three times daily for three minutes per session, using smartphone-based Ecological Momentary Assessment (EMA) to capture real-time pain outcomes."

1b-iii) Open vs. closed, web-based (self-assessment) vs. face-to-face assessments in the METHODS section of the ABSTRACT

Mention how participants were recruited (online vs. offline), e.g., from an open access website or from a clinic or a closed online user group (closed usergroup trial), and clarify if this was a purely web-based trial, or there were face-to-face components (as part of the intervention or for assessment). Clearly say if outcomes were self-assessed through questionnaires (as common in web-based trials). Note: In traditional offline trials, an open trial (open-label trial) is a type of clinical trial in which both the researchers and participants know which treatment is being administered. To avoid confusion, use “blinded” or “unblinded” to indicated the level of blinding instead of “open”, as “open” in web-based trials usually refers to “open access” (i.e. participants can self-enrol). (Note: Only report in the abstract what the main paper is reporting. If this information is missing from the main body of text, consider adding it)

	1	2	3	4	5	
subitem not at all important	○	○	●	○	○	essential

Clear selection

Does your paper address subitem 1b-iii?

Copy and paste relevant sections from the manuscript abstract (include quotes in quotation marks "like this" to indicate direct quotes from your manuscript), or elaborate on this item by providing additional information not in the ms, or briefly explain why the item is not applicable/relevant for your study

"These participants were recruited from outpatient oncology clinics, community engagement program, social media, and website."

1b-iv) RESULTS section in abstract must contain use data

Report number of participants enrolled/assessed in each group, the use/uptake of the intervention (e.g., attrition/adherence metrics, use over time, number of logins etc.), in addition to primary/secondary outcomes. (Note: Only report in the abstract what the main paper is reporting. If this information is missing from the main body of text, consider adding it)

	1	2	3	4	5	
subitem not at all important	○	○	○	○	◉	essential

Clear selection

Does your paper address subitem 1b-iv?

Copy and paste relevant sections from the manuscript abstract (include quotes in quotation marks "like this" to indicate direct quotes from your manuscript), or elaborate on this item by providing additional information not in the ms, or briefly explain why the item is not applicable/relevant for your study

The paper reporting the main outcomes and feasibility of the intervention was published elsewhere (https://doi.org/10.1093/pm/pnaf035). This paper aims to report the results of the smartphone-based Ecological Momentary Assessment (EMA), which is a crucial component of the intervention. We specifically report the compliance rate for the EMA component, as shown below.

"The study found an EMA compliance was 44.8%, with an attrition rate of 54%. No significant differences in compliance or attrition were observed across treatment arms. Compliance was significantly lower in older participants but was not associated with attrition. "

1b-v) CONCLUSIONS/DISCUSSION in abstract for negative trials

Conclusions/Discussions in abstract for negative trials: Discuss the primary outcome - if the trial is negative (primary outcome not changed), and the intervention was not used, discuss whether negative results are attributable to lack of uptake and discuss reasons. (Note: Only report in the abstract what the main paper is reporting. If this information is missing from the main body of text, consider adding it)

	1	2	3	4	5	
subitem not at all important	○	◉	○	○	○	essential

Clear selection

Does your paper address subitem 1b-v?

Copy and paste relevant sections from the manuscript abstract (include quotes in quotation marks "like this" to indicate direct quotes from your manuscript), or elaborate on this item by providing additional information not in the ms, or briefly explain why the item is not applicable/relevant for your study

The results demonstrated positive outcomes and promising evidence of APA's effectiveness. Therefore, our discussion emphasizes the factors that may have contributed to these improved results, as well as future directions, as outlined below.

"Conclusions: EMA data support APA as an effective pain management strategy for older adults with cLBP. Notably, optimal treatment efficacy was achieved with pressing durations ≥3 minutes, highlighting the importance of sufficient stimulation time. Future studies should refine APA regimens and improve EMA adherence strategies."

INTRODUCTION

2a) In INTRODUCTION: Scientific background and explanation of rationale

2a-i) Problem and the type of system/solution

Describe the problem and the type of system/solution that is object of the study: intended as stand-alone intervention vs. incorporated in broader health care program? Intended for a particular patient population? Goals of the intervention, e.g., being more cost-effective to other interventions, replace or complement other solutions? (Note: Details about the intervention are provided in “Methods” under 5)

	1	2	3	4	5	
subitem not at all important	○	○	○	○	●	essential

Clear selection

Does your paper address subitem 2a-i? *

Copy and paste relevant sections from the manuscript (include quotes in quotation marks "like this" to indicate direct quotes from your manuscript), or elaborate on this item by providing additional information not in the ms, or briefly explain why the item is not applicable/relevant for your study

"Moreover, by capturing the cyclical fluctuations of pain throughout the day, this assessment approach holds significant promise for enabling timely and personalized adjustments in pain management plans."

"APA is noted for its safety, simplicity, and potential to effectively reduce both pain and healthcare costs, integrating seamlessly into daily life [4-6]. However, the effect of APA on real-time pain outcomes is unclear."

"Therefore, we integrated APA with smartphone-based EMA to monitor pain outcomes in real-time, facilitating timely and personalized adjustments to pain management strategies."

2a-ii) Scientific background, rationale: What is known about the (type of) system

Scientific background, rationale: What is known about the (type of) system that is the object of the study (be sure to discuss the use of similar systems for other conditions/diagnoses, if appropiate), motivation for the study, i.e. what are the reasons for and what is the context for this specific study, from which stakeholder viewpoint is the study performed, potential impact of findings [2]. Briefly justify the choice of the comparator.

	1	2	3	4	5	
subitem not at all important	○	○	○	○	◉	essential

Clear selection

Does your paper address subitem 2a-ii? *

Copy and paste relevant sections from the manuscript (include quotes in quotation marks "like this" to indicate direct quotes from your manuscript), or elaborate on this item by providing additional information not in the ms, or briefly explain why the item is not applicable/relevant for your study

We explained the role of EMA and APA and highlight their importance for older adults with chronic low back pain. We also discussed previous studies on similar systems and how our study differs from them.

“Therefore, we integrated APA with smartphone-based EMA to monitor pain outcomes in real-time, facilitating timely and personalized adjustments to pain management strategies. Lin and colleagues (2019) has previously conducted a pilot study to evaluate the feasibility and usability of a smartphone application for real-time data collection on cLBP during a 4-week APA intervention. The study supports the feasibility of using EMA to reduce recall bias and accurately capture pain dynamics. The results of 18 cLBP participants who received weekly APA treatments indicated an 87% adherence rate, high usability ratings, and significant positive correlations between recalled and momentary pain measures, showing improvements in pain intensity and interference [4].
However previous studies on EMA for pain assessment have been limited by small sample sizes (n = 18), reducing the generalizability of findings.”

2b) In INTRODUCTION: Specific objectives or hypotheses

Does your paper address CONSORT subitem 2b? *

Copy and paste relevant sections from the manuscript (include quotes in quotation marks "like this" to indicate direct quotes from your manuscript), or elaborate on this item by providing additional information not in the ms, or briefly explain why the item is not applicable/relevant for your study

"Specifically, we aimed to evaluate the effectiveness and usability of APA for managing cLBP in older adults using a larger sample size and using EMA for capturing real-time pain outcomes. This study aims to provide evidence on the benefits of APA in reducing pain intensity and improving daily functioning and quality of life while addressing limitations related to recall bias through real-time data collection."

METHODS

3a) Description of trial design (such as parallel, factorial) including allocation ratio

Does your paper address CONSORT subitem 3a? *

Copy and paste relevant sections from the manuscript (include quotes in quotation marks "like this" to indicate direct quotes from your manuscript), or elaborate on this item by providing additional information not in the ms, or briefly explain why the item is not applicable/relevant for your study

"This study is a randomized controlled trial (1:1:1), evaluating the effectiveness of APA for managing cLBP in older adults, using smartphone EMA and ear APA to capture real-time pain outcomes."

3b) Important changes to methods after trial commencement (such as eligibility criteria), with reasons

Does your paper address CONSORT subitem 3b? *

Copy and paste relevant sections from the manuscript (include quotes in quotation marks "like this" to indicate direct quotes from your manuscript), or elaborate on this item by providing additional information not in the ms, or briefly explain why the item is not applicable/relevant for your study

There were no important changes to the methods, eligibility criteria, or other study procedures after trial commencement.

3b-i) Bug fixes, Downtimes, Content Changes

Bug fixes, Downtimes, Content Changes: ehealth systems are often dynamic systems. A description of changes to methods therefore also includes important changes made on the intervention or comparator during the trial (e.g., major bug fixes or changes in the functionality or content) (5-iii) and other “unexpected events” that may have influenced study design such as staff changes, system failures/downtimes, etc. [2].

	1	2	3	4	5	
subitem not at all important	○	○	○	○	◉	essential

Clear selection

Does your paper address subitem 3b-i?

Copy and paste relevant sections from the manuscript (include quotes in quotation marks "like this" to indicate direct quotes from your manuscript), or elaborate on this item by providing additional information not in the ms, or briefly explain why the item is not applicable/relevant for your study

"This study used a smartphone app named EarAPA, which was a revised version of the Earpress application [4] to collect the APA outcomes for cLBP. The application ran on the HTC Desire smartphone (Desire 612; HTC Corporation, New Taipei City, Taiwan). The app was revised and customized to enhance user-friendliness, including an enlarged font size and volume control to optimize use for older adults."

4a) Eligibility criteria for participants

Does your paper address CONSORT subitem 4a? *

Copy and paste relevant sections from the manuscript (include quotes in quotation marks "like this" to indicate direct quotes from your manuscript), or elaborate on this item by providing additional information not in the ms, or briefly explain why the item is not applicable/relevant for your study

"Individuals aged 60 years of age or older who were diagnosed with cLBP were enrolled. The inclusion criteria included 1) being able to read and write in English; 2) having cLBP that has persisted for at least 3 months, with pain occurring on at least half of the days over the previous 6 months [9]; 3) reporting an average pain intensity of 4 or higher on an 11-point numerical pain scale in the previous week and have intact cognition (Mini-Mental State Examination (MMSE) score > 24); 4) being able to apply pressure to the seeds with tapes on their ears; and 5) no new medication or pain-related treatments will be added during the 4-week intervention duration. Exclusion criteria for the study include the presence of malignant or autoimmune diseases (e.g., rheumatoid arthritis) where the pain cannot be distinguished from low back pain, known acute compression fractures caused by conditions like osteoporosis or spinal stenosis, and sciatica with leg pain greater than back pain. Participants with allergies to the tape used in the intervention, those using certain types of hearing aids that obstruct seed placement, or those experiencing more severe pain in other body parts that occurs daily or with moderate intensity are also excluded. Additionally, individuals with neurological disorders that could interfere with pain reporting, such as cerebral tumors, Alzheimer's disease, prior strokes, or multiple sclerosis, are not eligible to participate."

4a-i) Computer / Internet literacy

Computer / Internet literacy is often an implicit “de facto” eligibility criterion - this should be explicitly clarified.

	1	2	3	4	5	
subitem not at all important	○	○	●	○	○	essential

Clear selection

Does your paper address subitem 4a-i?

Copy and paste relevant sections from the manuscript (include quotes in quotation marks "like this" to indicate direct quotes from your manuscript), or elaborate on this item by providing additional information not in the ms, or briefly explain why the item is not applicable/relevant for your study

"Basic smartphone usage (e.g., opening apps, responding to prompts) was expected but not formally assessed."

4a-ii) Open vs. closed, web-based vs. face-to-face assessments:

Open vs. closed, web-based vs. face-to-face assessments: Mention how participants were recruited (online vs. offline), e.g., from an open access website or from a clinic, and clarify if this was a purely web-based trial, or there were face-to-face components (as part of the intervention or for assessment), i.e., to what degree got the study team to know the participant. In online-only trials, clarify if participants were quasi-anonymous and whether having multiple identities was possible or whether technical or logistical measures (e.g., cookies, email confirmation, phone calls) were used to detect/prevent these.

	1	2	3	4	5	
subitem not at all important	○	○	○	○	◉	essential

Clear selection

Does your paper address subitem 4a-ii? *

Copy and paste relevant sections from the manuscript (include quotes in quotation marks "like this" to indicate direct quotes from your manuscript), or elaborate on this item by providing additional information not in the ms, or briefly explain why the item is not applicable/relevant for your study

"1.3. Recruitment
Participants were recruited from outpatient clinics at Johns Hopkins Hospital, including the Pain Treatment Center, Community Physicians, Geriatric Medicine, and General Internal Medicine Clinics. Additional recruitment was conducted through the Community Engagement Program, social media platforms (e.g., Craigslist), and the ClinicalTrials.gov website. The participant registry maintained by the Healthy Aging Studies Unit at the Johns Hopkins Older Americans Independence Center was also utilized. Informed consent was obtained by trained study coordinators."

4a-iii) Information giving during recruitment

Information given during recruitment. Specify how participants were briefed for recruitment and in the informed consent procedures (e.g., publish the informed consent documentation as appendix, see also item X26), as this information may have an effect on user self-selection, user expectation and may also bias results.

	1	2	3	4	5	
subitem not at all important	○	◉	○	○	○	essential

Clear selection

Does your paper address subitem 4a-iii?

Copy and paste relevant sections from the manuscript (include quotes in quotation marks "like this" to indicate direct quotes from your manuscript), or elaborate on this item by providing additional information not in the ms, or briefly explain why the item is not applicable/relevant for your study

"Participants were fully informed during recruitment about eligibility, study purpose, their responsibilities, the study schedule, rights, and their understanding was confirmed before proceeding with consent."

4b) Settings and locations where the data were collected

Does your paper address CONSORT subitem 4b? *

Copy and paste relevant sections from the manuscript (include quotes in quotation marks "like this" to indicate direct quotes from your manuscript), or elaborate on this item by providing additional information not in the ms, or briefly explain why the item is not applicable/relevant for your study

The full details of the intervention and data collection procedures are described in our published protocol. Therefore, we have provided a summary in the manuscript, but readers can refer to the protocol for more comprehensive information.

4b-i) Report if outcomes were (self-)assessed through online questionnaires

Clearly report if outcomes were (self-)assessed through online questionnaires (as common in web-based trials) or otherwise.

	1	2	3	4	5	
subitem not at all important	○	○	○	○	◉	essential

Clear selection

Does your paper address subitem 4b-i? *

Copy and paste relevant sections from the manuscript (include quotes in quotation marks "like this" to indicate direct quotes from your manuscript), or elaborate on this item by providing additional information not in the ms, or briefly explain why the item is not applicable/relevant for your study

"The EarAPA app employed EMA to capture real-time data on pain intensity and daily interference due to cLBP over a specific period of 29 days. This concise timeframe enables a focused analysis of immediate treatment effects and short-term trends. The EarAPA app included two types of surveys for momentary pain assessment: (1) random EMA surveys to capture real-time pain outcomes and (2) time-contingent EMA surveys to monitor pain, APA practice adherence, and analgesic use, scheduled based on the participant's daily routine. The random EMA survey was prompted once daily during waking hours, asking four short questions about momentary pain intensity and pain interference with enjoyment of life and daily/general activity, which could be completed in about one minute. Time-contingent EMA surveys were prompted twice daily, additionally assessed APA practice, and analgesic use, taking less than two minutes to complete.
Survey questions appeared one at a time on the smartphone screen. Data from the EarAPA app were automatically uploaded in real time to a secure project website and were reviewed every 72 hours to track participation. If a participant failed to submit EMA data for more than two days, they were contacted by phone to identify and resolve any issues and were encouraged to resume completing the surveys."

4b-ii) Report how institutional affiliations are displayed

Report how institutional affiliations are displayed to potential participants [on ehealth media], as affiliations with prestigious hospitals or universities may affect volunteer rates, use, and reactions with regards to an intervention.(Not a required item – describe only if this may bias results)

	1	2	3	4	5	
subitem not at all important	○	◉	○	○	○	essential

Clear selection

Does your paper address subitem 4b-ii?

Copy and paste relevant sections from the manuscript (include quotes in quotation marks "like this" to indicate direct quotes from your manuscript), or elaborate on this item by providing additional information not in the ms, or briefly explain why the item is not applicable/relevant for your study

Institutional affiliations were displayed to participants through recruitment materials and consent forms, identifying the study as being conducted by Johns Hopkins University. Given the university's strong reputation, it is possible that this affiliation may have positively influenced volunteer interest and participation. However, the mobile application used for EMA did not display any institutional affiliation. We did not formally assess the impact of institutional affiliation on recruitment or participant responses.

5) The interventions for each group with sufficient details to allow replication, including how and when they were actually administered

5-i) Mention names, credential, affiliations of the developers, sponsors, and owners

Mention names, credential, affiliations of the developers, sponsors, and owners [6] (if authors/evaluators are owners or developer of the software, this needs to be declared in a “Conflict of interest” section or mentioned elsewhere in the manuscript).

	1	2	3	4	5	
subitem not at all important	○	◉	○	○	○	essential

Clear selection

Does your paper address subitem 5-i?

Copy and paste relevant sections from the manuscript (include quotes in quotation marks "like this" to indicate direct quotes from your manuscript), or elaborate on this item by providing additional information not in the ms, or briefly explain why the item is not applicable/relevant for your study

The mobile application was developed by Chichih Chen, who has no conflicts of interest related to this study. The funder of the study was mentioned in the Acknowledgement section of the manuscript. Neither the developers nor the sponsors had any ownership or financial interest in the study outcomes.

5-ii) Describe the history/development process

Describe the history/development process of the application and previous formative evaluations (e.g., focus groups, usability testing), as these will have an impact on adoption/use rates and help with interpreting results.

	1	2	3	4	5	
subitem not at all important	○	◉	○	○	○	essential

Clear selection

Does your paper address subitem 5-ii?

Copy and paste relevant sections from the manuscript (include quotes in quotation marks "like this" to indicate direct quotes from your manuscript), or elaborate on this item by providing additional information not in the ms, or briefly explain why the item is not applicable/relevant for your study

The history and development process of the mobile application, including previous formative evaluations and quality of information provided, have been published elsewhere. Relevant details are available in the cited publication DOI: 10.1177/2164956120987531

5-iii) Revisions and updating

Revisions and updating. Clearly mention the date and/or version number of the application/intervention (and comparator, if applicable) evaluated, or describe whether the intervention underwent major changes during the evaluation process, or whether the development and/or content was “frozen” during the trial. Describe dynamic components such as news feeds or changing content which may have an impact on the replicability of the intervention (for unexpected events see item 3b).

	1	2	3	4	5	
subitem not at all important	○	○	○	◉	○	essential

Clear selection

Does your paper address subitem 5-iii?

Copy and paste relevant sections from the manuscript (include quotes in quotation marks "like this" to indicate direct quotes from your manuscript), or elaborate on this item by providing additional information not in the ms, or briefly explain why the item is not applicable/relevant for your study

"The app was revised and customized to enhance user-friendliness, including an enlarged font size and volume control to optimize use for older adults."

5-iv) Quality assurance methods

Provide information on quality assurance methods to ensure accuracy and quality of information provided [1], if applicable.

	1	2	3	4	5	
subitem not at all important	○	◉	○	○	○	essential

Clear selection

Does your paper address subitem 5-iv?

Copy and paste relevant sections from the manuscript (include quotes in quotation marks "like this" to indicate direct quotes from your manuscript), or elaborate on this item by providing additional information not in the ms, or briefly explain why the item is not applicable/relevant for your study

The history and development process of the mobile application, including previous formative evaluations and quality of information provided, have been published elsewhere. Relevant details are available in the cited publication DOI: 10.1177/2164956120987531

5-v) Ensure replicability by publishing the source code, and/or providing screenshots/screen-capture video, and/or providing flowcharts of the algorithms used

Ensure replicability by publishing the source code, and/or providing screenshots/screen-capture video, and/or providing flowcharts of the algorithms used. Replicability (i.e., other researchers should in principle be able to replicate the study) is a hallmark of scientific reporting.

	1	2	3	4	5	
subitem not at all important	○	◉	○	○	○	essential

Clear selection

Does your paper address subitem 5-v?

Copy and paste relevant sections from the manuscript (include quotes in quotation marks "like this" to indicate direct quotes from your manuscript), or elaborate on this item by providing additional information not in the ms, or briefly explain why the item is not applicable/relevant for your study

This study aimed to evaluate the intervention delivered via EMA, which represents one component of the EarAPA app. Therefore, we did not focus on detailing the full mobile app intervention within this manuscript. Information about the app's development, including screenshots and further technical details, has been published elsewhere and is cited accordingly: DOI: 10.1177/2164956120987531.

5-vi) Digital preservation

Digital preservation: Provide the URL of the application, but as the intervention is likely to change or disappear over the course of the years; also make sure the intervention is archived (Internet Archive, webcitation.org, and/or publishing the source code or screenshots/videos alongside the article). As pages behind login screens cannot be archived, consider creating demo pages which are accessible without login.

	1	2	3	4	5	
subitem not at all important	○	◉	○	○	○	essential

Clear selection

Does your paper address subitem 5-vi?

Copy and paste relevant sections from the manuscript (include quotes in quotation marks "like this" to indicate direct quotes from your manuscript), or elaborate on this item by providing additional information not in the ms, or briefly explain why the item is not applicable/relevant for your study

The EarAPA app was made available on the app store (Android or IOS), but access is restricted to authorized participants.

5-vii) Access

Access: Describe how participants accessed the application, in what setting/context, if they had to pay (or were paid) or not, whether they had to be a member of specific group. If known, describe how participants obtained “access to the platform and Internet” [1]. To ensure access for editors/reviewers/readers, consider to provide a “backdoor” login account or demo mode for reviewers/readers to explore the application (also important for archiving purposes, see vi).

	1	2	3	4	5	
subitem not at all important	○	◉	○	○	○	essential

Clear selection

Does your paper address subitem 5-vii? *

Copy and paste relevant sections from the manuscript (include quotes in quotation marks "like this" to indicate direct quotes from your manuscript), or elaborate on this item by providing additional information not in the ms, or briefly explain why the item is not applicable/relevant for your study

The EarAPA app was made available on the app store (Android or IOS), but access is restricted to authorized participants.

5-viii) Mode of delivery, features/functionalities/components of the intervention and comparator, and the theoretical framework

Describe mode of delivery, features/functionalities/components of the intervention and comparator, and the theoretical framework [6] used to design them (instructional strategy [1], behaviour change techniques, persuasive features, etc., see e.g., [7, 8] for terminology). This includes an in-depth description of the content (including where it is coming from and who developed it) [1]," whether [and how] it is tailored to individual circumstances and allows users to track their progress and receive feedback" [6]. This also includes a description of communication delivery channels and – if computer-mediated communication is a component – whether communication was synchronous or asynchronous [6]. It also includes information on presentation strategies [1], including page design principles, average amount of text on pages, presence of hyperlinks to other resources, etc. [1].

	1	2	3	4	5	
subitem not at all important	○	○	◉	○	○	essential

Clear selection

Does your paper address subitem 5-viii? *

Copy and paste relevant sections from the manuscript (include quotes in quotation marks "like this" to indicate direct quotes from your manuscript), or elaborate on this item by providing additional information not in the ms, or briefly explain why the item is not applicable/relevant for your study

"Each participant received a smartphone with the EarAPA app pre-installed, along with a phone charger. This study used a smartphone app named EarAPA, which was a revised version of the Earpress application [4] to collect the APA outcomes for cLBP. The application ran on the HTC Desire smartphone (Desire 612; HTC Corporation, New Taipei City, Taiwan). The app was revised and customized to enhance user-friendliness, including an enlarged font size and volume control to optimize use for older adults. The history and development process of the mobile application, including previous formative evaluations, have been published elsewhere [12]. The EarAPA app was made available on the app store (Android or IOS), but access is restricted to authorized participants."

The full information about the app has been published elsewhere and is cited accordingly: DOI: 10.1177/2164956120987531.

5-ix) Describe use parameters

Describe use parameters (e.g., intended “doses” and optimal timing for use). Clarify what instructions or recommendations were given to the user, e.g., regarding timing, frequency, heaviness of use, if any, or was the intervention used ad libitum.

	1	2	3	4	5	
subitem not at all important	○	○	○	◉	○	essential

Clear selection

Does your paper address subitem 5-ix?

Copy and paste relevant sections from the manuscript (include quotes in quotation marks "like this" to indicate direct quotes from your manuscript), or elaborate on this item by providing additional information not in the ms, or briefly explain why the item is not applicable/relevant for your study

"The EarAPA app employed EMA to capture real-time data on pain intensity and daily interference due to cLBP over a specific period of 29 days. This concise timeframe enables a focused analysis of immediate treatment effects and short-term trends. The EarAPA app included two types of surveys for momentary pain assessment: (1) random EMA surveys to capture real-time pain outcomes and (2) time-contingent EMA surveys to monitor pain, APA practice adherence, and analgesic use, scheduled based on the participant's daily routine. The random EMA survey was prompted once daily during waking hours, asking four short questions about momentary pain intensity and pain interference with enjoyment of life and daily/general activity, which could be completed in about one minute. Time-contingent EMA surveys were prompted twice daily, additionally assessed APA practice, and analgesic use, taking less than two minutes to complete."

5-x) Clarify the level of human involvement

Clarify the level of human involvement (care providers or health professionals, also technical assistance) in the e-intervention or as co-intervention (detail number and expertise of professionals involved, if any, as well as “type of assistance offered, the timing and frequency of the support, how it is initiated, and the medium by which the assistance is delivered”. It may be necessary to distinguish between the level of human involvement required for the trial, and the level of human involvement required for a routine application outside of a RCT setting (discuss under item 21 – generalizability).

	1	2	3	4	5	
subitem not at all important	○	○	◉	○	○	essential

Clear selection

Does your paper address subitem 5-x?

Copy and paste relevant sections from the manuscript (include quotes in quotation marks "like this" to indicate direct quotes from your manuscript), or elaborate on this item by providing additional information not in the ms, or briefly explain why the item is not applicable/relevant for your study

"The APA intervention involved the self-application of small seeds at specific auricular points, which participants were instructed to press multiple times a day. For the T-APA group, points specific to alleviating cLBP were targeted including lower back, nervous subcortex, sympathetic, and Shenmen, while the NT-APA group received acupressure at points related to the mouth, stomach, duodenum, internal ear, and tonsil - areas not associated with cLBP [10, 11]. The education control group received an educational booklet detailing strategies for managing back pain. All participants received weekly sessions over four weeks, each involving the application of new seeds and a review of the technique by the practitioners. Additionally, all participants were provided with a smartphone application (EarAPA) to collect real-time cLBP outcomes and monitor adherence to APA practice."

5-xi) Report any prompts/reminders used

Report any prompts/reminders used: Clarify if there were prompts (letters, emails, phone calls, SMS) to use the application, what triggered them, frequency etc. It may be necessary to distinguish between the level of prompts/reminders required for the trial, and the level of prompts/reminders for a routine application outside of a RCT setting (discuss under item 21 – generalizability).

	1	2	3	4	5	
subitem not at all important	○	○	○	○	◉	essential

Clear selection

Does your paper address subitem 5-xi? *

Copy and paste relevant sections from the manuscript (include quotes in quotation marks "like this" to indicate direct quotes from your manuscript), or elaborate on this item by providing additional information not in the ms, or briefly explain why the item is not applicable/relevant for your study

"The EarAPA app employed EMA to capture real-time data on pain intensity and daily interference due to cLBP over a specific period of 29 days. This concise timeframe enables a focused analysis of immediate treatment effects and short-term trends. The EarAPA app included two types of surveys for momentary pain assessment: (1) random EMA surveys to capture real-time pain outcomes and (2) time-contingent EMA surveys to monitor pain, APA practice adherence, and analgesic use, scheduled based on the participant's daily routine. The random EMA survey was prompted once daily during waking hours, asking four short questions about momentary pain intensity and pain interference with enjoyment of life and daily/general activity, which could be completed in about one minute. Time-contingent EMA surveys were prompted twice daily, additionally assessed APA practice, and analgesic use, taking less than two minutes to complete.
Survey questions appeared one at a time on the smartphone screen. Data from the EarAPA app were automatically uploaded in real time to a secure project website and were reviewed every 72 hours to track participation. If a participant failed to submit EMA data for more than two days, they were contacted by phone to identify and resolve any issues and were encouraged to resume completing the surveys."

5-xii) Describe any co-interventions (incl. training/support)

Describe any co-interventions (incl. training/support): Clearly state any interventions that are provided in addition to the targeted eHealth intervention, as ehealth intervention may not be designed as stand-alone intervention. This includes training sessions and support [1]. It may be necessary to distinguish between the level of training required for the trial, and the level of training for a routine application outside of a RCT setting (discuss under item 21 – generalizability.

	1	2	3	4	5	
subitem not at all important	○	○	○	◉	○	essential

Clear selection

Does your paper address subitem 5-xii? *

Copy and paste relevant sections from the manuscript (include quotes in quotation marks "like this" to indicate direct quotes from your manuscript), or elaborate on this item by providing additional information not in the ms, or briefly explain why the item is not applicable/relevant for your study

"The specifics of the APA treatment protocol are detailed extensively in the published protocol paper [7]. This includes precise locations of auricular points, frequency of treatments, and application techniques used by trained therapists. The APA intervention involved the self-application of small seeds at specific auricular points, which participants were instructed to press multiple times a day. For the T-APA group, points specific to alleviating cLBP were targeted including lower back, nervous subcortex, sympathetic, and Shenmen, while the NT-APA group received acupressure at points related to the mouth, stomach, duodenum, internal ear, and tonsil - areas not associated with cLBP [10, 11]. The education control group received an educational booklet detailing strategies for managing back pain. All participants received weekly sessions over four weeks, each involving the application of new seeds and a review of the technique by the practitioners. Additionally, all participants were provided with a smartphone application (EarAPA) to collect real-time cLBP outcomes and monitor adherence to APA practice. For detailed methodology and intervention procedures, please refer to the protocol publication [7]."

6a) Completely defined pre-specified primary and secondary outcome measures, including how and when they were assessed

Does your paper address CONSORT subitem 6a? *

Copy and paste relevant sections from the manuscript (include quotes in quotation marks "like this" to indicate direct quotes from your manuscript), or elaborate on this item by providing additional information not in the ms, or briefly explain why the item is not applicable/relevant for your study

EMA was used to evaluate the primary outcomes, including pain intensity and pain interference three times daily, while APA practice and analgesic use were assessed twice daily.

6a-i) Online questionnaires: describe if they were validated for online use and apply CHERRIES items to describe how the questionnaires were designed/deployed

If outcomes were obtained through online questionnaires, describe if they were validated for online use and apply CHERRIES items to describe how the questionnaires were designed/deployed [9].

	1	2	3	4	5	
subitem not at all important	○	◉	○	○	○	essential

Clear selection

Does your paper address subitem 6a-i?

Copy and paste relevant sections from manuscript text

We did not specifically follow the CHERRIES checklist for questionnaire design. Instead, we used EMA items that had been previously developed and used in earlier studies. The questions used to assess pain intensity, pain interference, APA practice, and analgesic use were adapted from prior published work to ensure consistency with established measures.

6a-ii) Describe whether and how “use” (including intensity of use/dosage) was defined/measured/monitored

Describe whether and how “use” (including intensity of use/dosage) was defined/measured/monitored (logins, logfile analysis, etc.). Use/adoption metrics are important process outcomes that should be reported in any ehealth trial.

	1	2	3	4	5	
subitem not at all important	○	○	○	○	○	essential

Does your paper address subitem 6a-ii?

Copy and paste relevant sections from manuscript text

In this study, “use” was monitored through self-reported EMA entries completed by participants using the mobile application. Intensity of use (i.e., dosage) was captured by participants' responses to twice-daily EMA prompts regarding their APA practice, specifically the number of times per day they pressed the seeds and the duration of pressing.

“The APA intervention involved the self-application of small seeds at specific auricular points, which participants were instructed to press then three minutes per session, three sessions a day.“

“The EarAPA app included two types of surveys for momentary pain assessment: (1) random EMA surveys to capture real-time pain outcomes and (2) time-contingent EMA surveys to monitor pain, APA practice adherence, and analgesic use, scheduled based on the participant's daily routine. The random EMA survey was prompted once daily during waking hours, asking four short questions about momentary pain intensity and pain interference with enjoyment of life and daily/general activity, which could be completed in about one minute.“

6a-iii) Describe whether, how, and when qualitative feedback from participants was obtained

Describe whether, how, and when qualitative feedback from participants was obtained (e.g., through emails, feedback forms, interviews, focus groups).

	1	2	3	4	5	
subitem not at all important	○	◉	○	○	○	essential

Clear selection

Does your paper address subitem 6a-iii?

Copy and paste relevant sections from manuscript text

Qualitative feedback regarding participants' experiences with the mobile application was collected and has been published separately. Readers are referred to the related publication for detailed findings on user feedback. https://doi.org/10.1177/2164956120987531

6b) Any changes to trial outcomes after the trial commenced, with reasons

Does your paper address CONSORT subitem 6b? *

Copy and paste relevant sections from the manuscript (include quotes in quotation marks "like this" to indicate direct quotes from your manuscript), or elaborate on this item by providing additional information not in the ms, or briefly explain why the item is not applicable/relevant for your study

There were no changes to the trial outcomes after the trial commenced.

7a) How sample size was determined

NPT: When applicable, details of whether and how the clustering by care provides or centers was addressed

7a-i) Describe whether and how expected attrition was taken into account when calculating the sample size

Describe whether and how expected attrition was taken into account when calculating the sample size.

	1	2	3	4	5	
subitem not at all important	○	●	○	○	○	essential

Clear selection

Does your paper address subitem 7a-i?

Copy and paste relevant sections from manuscript title (include quotes in quotation marks "like this" to indicate direct quotes from your manuscript), or elaborate on this item by providing additional information not in the ms, or briefly explain why the item is not applicable/relevant for your study

This study presents additional results from an existing clinical trial. The sample size calculation has been published elsewhere (https://doi.org/10.1093/pm/pnaf035).

7b) When applicable, explanation of any interim analyses and stopping guidelines

Does your paper address CONSORT subitem 7b? *

Copy and paste relevant sections from the manuscript (include quotes in quotation marks "like this" to indicate direct quotes from your manuscript), or elaborate on this item by providing additional information not in the ms, or briefly explain why the item is not applicable/relevant for your study

There were no interim analyses or stopping guidelines planned or conducted for this study, as outlined in the study protocol.

8a) Method used to generate the random allocation sequence
NPT: When applicable, how care providers were allocated to each trial group

Does your paper address CONSORT subitem 8a? *

Copy and paste relevant sections from the manuscript (include quotes in quotation marks "like this" to indicate direct quotes from your manuscript), or elaborate on this item by providing additional information not in the ms, or briefly explain why the item is not applicable/relevant for your study

"Participants were randomly assigned to one of the three study groups (1:1:1 ratio) : targeted APA (T-APA), non-targeted APA (NT-APA), or education control, using a computer-generated sequence to ensure an unbiased distribution."

8b) Type of randomisation; details of any restriction (such as blocking and block size)

Does your paper address CONSORT subitem 8b? *

Copy and paste relevant sections from the manuscript (include quotes in quotation marks "like this" to indicate direct quotes from your manuscript), or elaborate on this item by providing additional information not in the ms, or briefly explain why the item is not applicable/relevant for your study

"Randomization was conducted in blocks of three or six, with participants evenly allocated across the three groups based on the anticipated number of eligible participants."

9) Mechanism used to implement the random allocation sequence (such as sequentially numbered containers), describing any steps taken to conceal the sequence until interventions were assigned

Does your paper address CONSORT subitem 9? *

Copy and paste relevant sections from the manuscript (include quotes in quotation marks "like this" to indicate direct quotes from your manuscript), or elaborate on this item by providing additional information not in the ms, or briefly explain why the item is not applicable/relevant for your study

"Participants were randomly assigned to one of the three study groups (1:1:1 ratio) : targeted APA (T-APA), non-targeted APA (NT-APA), or education control, using a computer-generated sequence to ensure an unbiased distribution."

10) Who generated the random allocation sequence, who enrolled participants, and who assigned participants to interventions

Does your paper address CONSORT subitem 10? *

Copy and paste relevant sections from the manuscript (include quotes in quotation marks "like this" to indicate direct quotes from your manuscript), or elaborate on this item by providing additional information not in the ms, or briefly explain why the item is not applicable/relevant for your study

"Participants were randomly assigned to one of the three study groups (1:1:1 ratio) : targeted APA (T-APA), non-targeted APA (NT-APA), or education control, using a computer-generated sequence to ensure an unbiased distribution."

11a) If done, who was blinded after assignment to interventions (for example, participants, care providers, those assessing outcomes) and how

NPT: Whether or not administering co-interventions were blinded to group assignment

11a-i) Specify who was blinded, and who wasn't

Specify who was blinded, and who wasn't. Usually, in web-based trials it is not possible to blind the participants [1, 3] (this should be clearly acknowledged), but it may be possible to blind outcome assessors, those doing data analysis or those administering co-interventions (if any).

	1	2	3	4	5	
subitem not at all important	○	◉	○	○	○	essential

Clear selection

Does your paper address subitem 11a-i? *

Copy and paste relevant sections from the manuscript (include quotes in quotation marks "like this" to indicate direct quotes from your manuscript), or elaborate on this item by providing additional information not in the ms, or briefly explain why the item is not applicable/relevant for your study

"Participants in the APA groups were blinded to their group assignment. Additionally, co-investigators, data collectors, and analysts remained blinded to participant group allocations."

11a-ii) Discuss e.g., whether participants knew which intervention was the "intervention of interest" and which one was the "comparator"

Informed consent procedures (4a-ii) can create biases and certain expectations - discuss e.g., whether participants knew which intervention was the "intervention of interest" and which one was the "comparator".

	1	2	3	4	5	
subitem not at all important	○	○	○	◉	○	essential

Clear selection

Does your paper address subitem 11a-ii?

Copy and paste relevant sections from the manuscript (include quotes in quotation marks "like this" to indicate direct quotes from your manuscript), or elaborate on this item by providing additional information not in the ms, or briefly explain why the item is not applicable/relevant for your study

Participants were blinded to their group assignments and were not informed which intervention was considered the primary intervention of interest (targeted APA) and which served as the comparator (non-targeted APA).

11b) If relevant, description of the similarity of interventions

(this item is usually not relevant for ehealth trials as it refers to similarity of a placebo or sham intervention to a active medication/intervention)

Does your paper address CONSORT subitem 11b? *

Copy and paste relevant sections from the manuscript (include quotes in quotation marks "like this" to indicate direct quotes from your manuscript), or elaborate on this item by providing additional information not in the ms, or briefly explain why the item is not applicable/relevant for your study

"For the T-APA group, points specific to alleviating cLBP were targeted including lower back, nervous subcortex, sympathetic, and Shenmen, while the NT-APA group received acupressure at points related to the mouth, stomach, duodenum, internal ear, and tonsil - areas not associated with cLBP [10, 11]."

12a) Statistical methods used to compare groups for primary and secondary outcomes

NPT: When applicable, details of whether and how the clustering by care providers or centers was addressed

Does your paper address CONSORT subitem 12a? *

Copy and paste relevant sections from the manuscript (include quotes in quotation marks "like this" to indicate direct quotes from your manuscript), or elaborate on this item by providing additional information not in the ms, or briefly explain why the item is not applicable/relevant for your study

"A linear mixed-effects model was used to assess the influence of APA treatment on the longitudinal change in pain intensity and pain interference with EMA observation clustered within participants. The fixed effects included treatment group (T-APA, NT-APA, and education control), APA practice (pressing frequency, minutes per pressing, and total pressing time), analgesic use, treatment days (post-baseline), and covariates such as age, gender, race, smoking status, employment status, baseline opioid use, worst pain, average pain, current pain, pain interference with enjoyment of life, and pain interference with daily activities, all measured at baseline. Random effects included random intercepts and slopes for APA practice and analgesic use. A covariance matrix with a first-order autoregressive structure was used to model temporal correlations in the longitudinal data. Note that total pressing time per day was calculated as pressing frequency on that day multiplied by minutes per pressing. The linear mixed model can handle missing data under the assumption of missing at random."

12a-i) Imputation techniques to deal with attrition / missing values

Imputation techniques to deal with attrition / missing values: Not all participants will use the intervention/comparator as intended and attrition is typically high in ehealth trials. Specify how participants who did not use the application or dropped out from the trial were treated in the statistical analysis (a complete case analysis is strongly discouraged, and simple imputation techniques such as LOCF may also be problematic [4]).

	1	2	3	4	5	
subitem not at all important	○	◉	○	○	○	essential

Clear selection

Does your paper address subitem 12a-i? *

Copy and paste relevant sections from the manuscript (include quotes in quotation marks "like this" to indicate direct quotes from your manuscript), or elaborate on this item by providing additional information not in the ms, or briefly explain why the item is not applicable/relevant for your study

"The linear mixed model can handle missing data under the assumption of missing at random"

12b) Methods for additional analyses, such as subgroup analyses and adjusted analyses

Does your paper address CONSORT subitem 12b? *

Copy and paste relevant sections from the manuscript (include quotes in quotation marks "like this" to indicate direct quotes from your manuscript), or elaborate on this item by providing additional information not in the ms, or briefly explain why the item is not applicable/relevant for your study

"Multiple testing adjustments were not performed for this exploratory data analysis."

X26) REB/IRB Approval and Ethical Considerations [recommended as subheading under "Methods"] (not a CONSORT item)

X26-i) Comment on ethics committee approval

	1	2	3	4	5	
subitem not at all important	○	○	○	○	◉	essential

Clear selection

Does your paper address subitem X26-i?

Copy and paste relevant sections from the manuscript (include quotes in quotation marks "like this" to indicate direct quotes from your manuscript), or elaborate on this item by providing additional information not in the ms, or briefly explain why the item is not applicable/relevant for your study

This study protocol was approved by the Johns Hopkins Medicine Institutional Review Board (IRB00175409).

x26-ii) Outline informed consent procedures

Outline informed consent procedures e.g., if consent was obtained offline or online (how? Checkbox, etc.?), and what information was provided (see 4a-ii). See [6] for some items to be included in informed consent documents.

	1	2	3	4	5	
subitem not at all important	○	○	●	○	○	essential

Clear selection

Does your paper address subitem X26-ii?

Copy and paste relevant sections from the manuscript (include quotes in quotation marks "like this" to indicate direct quotes from your manuscript), or elaborate on this item by providing additional information not in the ms, or briefly explain why the item is not applicable/relevant for your study

"Informed consent was obtained by trained study coordinators. Participants were fully informed during recruitment about eligibility, study purpose, their responsibilities, the study schedule, rights, and their understanding was confirmed before proceeding with consent."

X26-iii) Safety and security procedures

Safety and security procedures, incl. privacy considerations, and any steps taken to reduce the likelihood or detection of harm (e.g., education and training, availability of a hotline)

	1	2	3	4	5	
subitem not at all important	○	●	○	○	○	essential

Clear selection

Does your paper address subitem X26-iii?

Copy and paste relevant sections from the manuscript (include quotes in quotation marks "like this" to indicate direct quotes from your manuscript), or elaborate on this item by providing additional information not in the ms, or briefly explain why the item is not applicable/relevant for your study

The safety and security procedures, including privacy considerations and protocols to minimize risks, have been described in detail in a separate publication. Readers are referred to that source for full information: https://doi.org/10.1186/s13063-019-4016-x

RESULTS

13a) For each group, the numbers of participants who were randomly assigned, received intended treatment, and were analysed for the primary outcome

NPT: The number of care providers or centers performing the intervention in each group and the number of patients treated by each care provider in each center

Does your paper address CONSORT subitem 13a? *

Copy and paste relevant sections from the manuscript (include quotes in quotation marks "like this" to indicate direct quotes from your manuscript), or elaborate on this item by providing additional information not in the ms, or briefly explain why the item is not applicable/relevant for your study

"Out of 700 individuals assessed for eligibility, 272 were randomized into three groups: T-APA (n=92), NT-APA (n=91), and control (n=89). Most exclusions (n=428) were due to ineligibility, inability to be reached, or unwillingness to commit to 12 months. In the T-APA group, 84 received ≥2 sessions, and 81 completed 1-month follow-up. In the NT-APA group, 78 received ≥2 sessions, and 68 completed 1-month follow-up. In the control group, 80 received ≥2 sessions, and 71 completed 1-month follow-up. Final analyses included 72 in T-APA, 74 in NT-APA, and 65 in the control group, while 61 participants had no EMA data collected and were excluded from this analysis. The CONSORT diagram can be found in Figure 1."

13b) For each group, losses and exclusions after randomisation, together with reasons

Does your paper address CONSORT subitem 13b? (NOTE: Preferably, this is shown in a CONSORT flow diagram) *

Copy and paste relevant sections from the manuscript (include quotes in quotation marks "like this" to indicate direct quotes from your manuscript), or elaborate on this item by providing additional information not in the ms, or briefly explain why the item is not applicable/relevant for your study

"Out of 700 individuals assessed for eligibility, 272 were randomized into three groups: T-APA (n=92), NT-APA (n=91), and control (n=89). Most exclusions (n=428) were due to ineligibility, inability to be reached, or unwillingness to commit to 12 months. In the T-APA group, 84 received ≥2 sessions, and 81 completed 1-month follow-up. In the NT-APA group, 78 received ≥2 sessions, and 68 completed 1-month follow-up. In the control group, 80 received ≥2 sessions, and 71 completed 1-month follow-up. Final analyses included 72 in T-APA, 74 in NT-APA, and 65 in the control group, while 61 participants had no EMA data collected and were excluded from this analysis. The CONSORT diagram can be found in Figure 1."

13b-i) Attrition diagram

Strongly recommended: An attrition diagram (e.g., proportion of participants still logging in or using the intervention/comparator in each group plotted over time, similar to a survival curve) or other figures or tables demonstrating usage/dose/engagement.

	1	2	3	4	5	
subitem not at all important	○	○	○	○	◉	essential

Clear selection

Does your paper address subitem 13b-i?

Copy and paste relevant sections from the manuscript or cite the figure number if applicable (include quotes in quotation marks "like this" to indicate direct quotes from your manuscript), or elaborate on this item by providing additional information not in the ms, or briefly explain why the item is not applicable/relevant for your study

"1.1. EMA compliance and attrition
Based on the study design, the EMA measurements were scheduled 3 times per day from baseline (day 0) to the 28 days of treatment (a total of 87 measurements for 29 days). The compliance rate was 44.8%, and the attrition rate was 54% (Fig. 2) . There was no significant difference in compliance and attrition rates among the three treatment arms. Gender, race, analgesic use, opioid, and baseline pain measurements were not significantly related to compliance and attrition rates. However, age was significantly associated with compliance, with older participants demonstrating lower compliance rates (F = 6.96, df = 1,209, p-value = 0.0090), though it was not related to attrition (p-value = 0.188)."

14a) Dates defining the periods of recruitment and follow-up

Does your paper address CONSORT subitem 14a? *

Copy and paste relevant sections from the manuscript (include quotes in quotation marks "like this" to indicate direct quotes from your manuscript), or elaborate on this item by providing additional information not in the ms, or briefly explain why the item is not applicable/relevant for your study

"Recruitment took place from March 1, 2019, to March 13, 2020, was paused due to the COVID-19 pandemic, and resumed from June 12, 2021, to November 18, 2021. Final follow-up and data collection were completed by December 1, 2022."

14a-i) Indicate if critical "secular events" fell into the study period

Indicate if critical "secular events" fell into the study period, e.g., significant changes in Internet resources available or "changes in computer hardware or Internet delivery resources"

	1	2	3	4	5	
subitem not at all important	○	◉	○	○	○	essential

Clear selection

Does your paper address subitem 14a-i?

Copy and paste relevant sections from the manuscript (include quotes in quotation marks "like this" to indicate direct quotes from your manuscript), or elaborate on this item by providing additional information not in the ms, or briefly explain why the item is not applicable/relevant for your study

"Recruitment took place from March 1, 2019, to March 13, 2020, was paused due to the COVID-19 pandemic, and resumed from June 12, 2021, to November 18, 2021. Final follow-up and data collection were completed by December 1, 2022."

14b) Why the trial ended or was stopped (early)

Does your paper address CONSORT subitem 14b? *

Copy and paste relevant sections from the manuscript (include quotes in quotation marks "like this" to indicate direct quotes from your manuscript), or elaborate on this item by providing additional information not in the ms, or briefly explain why the item is not applicable/relevant for your study

The trial was not ended or stopped early.

15) A table showing baseline demographic and clinical characteristics for each group

NPT: When applicable, a description of care providers (case volume, qualification, expertise, etc.) and centers (volume) in each group

Does your paper address CONSORT subitem 15? *

Copy and paste relevant sections from the manuscript (include quotes in quotation marks "like this" to indicate direct quotes from your manuscript), or elaborate on this item by providing additional information not in the ms, or briefly explain why the item is not applicable/relevant for your study

"The demographics and baseline characteristics, summarized in Table 1, indicate that these factors are well-balanced across the three groups."

15-i) Report demographics associated with digital divide issues

In ehealth trials it is particularly important to report demographics associated with digital divide issues, such as age, education, gender, social-economic status, computer/Internet/ehealth literacy of the participants, if known.

	1	2	3	4	5	
subitem not at all important	○	◉	○	○	○	essential

Clear selection

Does your paper address subitem 15-i? *

Copy and paste relevant sections from the manuscript (include quotes in quotation marks "like this" to indicate direct quotes from your manuscript), or elaborate on this item by providing additional information not in the ms, or briefly explain why the item is not applicable/relevant for your study

"The demographics and baseline characteristics, summarized in Table 1, indicate that these factors are well-balanced across the three groups."

16) For each group, number of participants (denominator) included in each analysis and whether the analysis was by original assigned groups

16-i) Report multiple "denominators" and provide definitions

Report multiple "denominators" and provide definitions: Report N's (and effect sizes) "across a range of study participation [and use] thresholds" [1], e.g., N exposed, N consented, N used more than x times, N used more than y weeks, N participants "used" the intervention/comparator at specific pre-defined time points of interest (in absolute and relative numbers per group). Always clearly define "use" of the intervention.

	1	2	3	4	5	
subitem not at all important	○	○	○	◉	○	essential

Clear selection

Does your paper address subitem 16-i? *

Copy and paste relevant sections from the manuscript (include quotes in quotation marks "like this" to indicate direct quotes from your manuscript), or elaborate on this item by providing additional information not in the ms, or briefly explain why the item is not applicable/relevant for your study

"Final analyses included 72 in T-APA, 74 in NT-APA, and 65 in the control group, while 61 participants had no EMA data collected and were excluded from this analysis."

16-ii) Primary analysis should be intent-to-treat

Primary analysis should be intent-to-treat, secondary analyses could include comparing only “users”, with the appropriate caveats that this is no longer a randomized sample (see 18-i).

	1	2	3	4	5	
subitem not at all important	○	◉	○	○	○	essential

Clear selection

Does your paper address subitem 16-ii?

Copy and paste relevant sections from the manuscript (include quotes in quotation marks "like this" to indicate direct quotes from your manuscript), or elaborate on this item by providing additional information not in the ms, or briefly explain why the item is not applicable/relevant for your study

This subitem is not directly applicable to our study. Our analyses were conducted based on the original group assignments (intention-to-treat), and all randomized participants were included in the analysis using available EMA data. We did not perform stratified analyses based on different thresholds of intervention use or EMA completion rates. Therefore, we report results based on the full randomized sample rather than across varying levels of study engagement.

17a) For each primary and secondary outcome, results for each group, and the estimated effect size and its precision (such as 95% confidence interval)

Does your paper address CONSORT subitem 17a? *

Copy and paste relevant sections from the manuscript (include quotes in quotation marks "like this" to indicate direct quotes from your manuscript), or elaborate on this item by providing additional information not in the ms, or briefly explain why the item is not applicable/relevant for your study

For each primary and secondary outcome, results for each group, including estimated effect sizes and their precision (95% confidence intervals), are presented in the Results section of the manuscript.

17a-i) Presentation of process outcomes such as metrics of use and intensity of use

In addition to primary/secondary (clinical) outcomes, the presentation of process outcomes such as metrics of use and intensity of use (dose, exposure) and their operational definitions is critical. This does not only refer to metrics of attrition (13-b) (often a binary variable), but also to more continuous exposure metrics such as "average session length". These must be accompanied by a technical description how a metric like a "session" is defined (e.g., timeout after idle time) [1] (report under item 6a).

	1	2	3	4	5	
subitem not at all important	○	○	○	●	○	essential

Clear selection

Does your paper address subitem 17a-i?

Copy and paste relevant sections from the manuscript (include quotes in quotation marks "like this" to indicate direct quotes from your manuscript), or elaborate on this item by providing additional information not in the ms, or briefly explain why the item is not applicable/relevant for your study

Operational definitions of use metrics:
"APA practice was evaluated based on three factors: the frequency of pressing the taped seeds, minutes per pressing, and total pressing time. Analgesic use was assessed by asking participants to report the dosage and frequency of any pain medication taken each day."

Technical description of how data were collected:
"The EarAPA app included two types of surveys for momentary pain assessment: (1) random EMA surveys to capture real-time pain outcomes and (2) time-contingent EMA surveys to monitor pain, APA practice adherence, and analgesic use, scheduled based on the participant's daily routine. The random EMA survey was prompted once daily during waking hours, asking four short questions about momentary pain intensity and pain interference with enjoyment of life and daily/general activity, which could be completed in about one minute. Time-contingent EMA surveys were prompted twice daily, additionally assessed APA practice, and analgesic use, taking less than two minutes to complete. Survey questions appeared one at a time on the smartphone screen. Data from the EarAPA app were automatically uploaded in real time to a secure project website and were reviewed every 72 hours to track participation."

Process outcomes reported:
"The compliance rate was 44.8%, and the attrition rate was 54% (Fig. 2). There was no significant difference in compliance and attrition rates among the three treatment arms. Gender, race, analgesic use, opioid, and baseline pain measurements were not significantly related to compliance and attrition rates. However, age was significantly associated with compliance, with older participants demonstrating lower compliance rates (F = 6.96, df = 1,209, P-value = 0.0090), though it was not related to attrition (P-value = 0.188)."

Findings on intensity of use:
"For APA practice, longer time per pressing (more than 3 minutes) was associated with significantly reduced current pain. However, higher pressing frequency (more than 3 times per day) was associated with increased worst pain and average pain. Total pressing time (more than 9 minutes per day) was not significantly associated with pain intensity outcomes."

17b) For binary outcomes, presentation of both absolute and relative effect sizes is recommended

Does your paper address CONSORT subitem 17b? *

Copy and paste relevant sections from the manuscript (include quotes in quotation marks "like this" to indicate direct quotes from your manuscript), or elaborate on this item by providing additional information not in the ms, or briefly explain why the item is not applicable/relevant for your study

This item is not applicable to our study. Our study outcomes were continuous variables, including pain intensity and pain interference scores measured through Ecological Momentary Assessment (EMA) and 7-day recall. No binary outcomes (e.g., yes/no events) were analyzed or reported. Therefore, absolute and relative effect sizes for binary outcomes were not presented.

18) Results of any other analyses performed, including subgroup analyses and adjusted analyses, distinguishing pre-specified from exploratory

Does your paper address CONSORT subitem 18? *

Copy and paste relevant sections from the manuscript (include quotes in quotation marks "like this" to indicate direct quotes from your manuscript), or elaborate on this item by providing additional information not in the ms, or briefly explain why the item is not applicable/relevant for your study

Yes, our manuscript addresses this item.

Adjusted analyses were performed as pre-specified in our protocol.
We used linear mixed-effects models to analyze the effects of the intervention on pain intensity and pain interference outcomes, adjusting for baseline covariates including: "age, gender, race, smoking status, employment status, baseline opioid use, worst pain, average pain, current pain, pain interference with enjoyment of life, and pain interference with daily activities."

Results of adjusted analyses were reported in Tables 4 and 5:
"Both T-APA and NT-APA treatments, compared to control, as well as the length of treatment, significantly reduced pain intensity and pain interference outcomes."

Exploratory analyses included:
1) The relationship between APA practice variables (pressing frequency, minutes per pressing, and total pressing time) and pain outcomes.
2) Analysis of compliance and attrition patterns by demographic factors (e.g., age and compliance relationship).

18-i) Subgroup analysis of comparing only users

A subgroup analysis of comparing only users is not uncommon in ehealth trials, but if done, it must be stressed that this is a self-selected sample and no longer an unbiased sample from a randomized trial (see 16-iii).

	1	2	3	4	5	
subitem not at all important	○	○	○	◉	○	essential

Clear selection

Does your paper address subitem 18-i?

Copy and paste relevant sections from the manuscript (include quotes in quotation marks "like this" to indicate direct quotes from your manuscript), or elaborate on this item by providing additional information not in the ms, or briefly explain why the item is not applicable/relevant for your study

Yes, our manuscript includes a subgroup analysis comparing participants who completed EMA surveys ("EMA users").

We acknowledged that participants who had EMA data differed from those without EMA data:
"Participants without EMA were significantly older than those with EMA (72.15 vs. 69.36 years, p = 0.0056), and those with EMA reported greater pain interference with enjoyment of life (4.74 vs. 3.80, p = 0.0409)."

We limited the primary EMA analyses to participants who had EMA data (n=211).

19) All important harms or unintended effects in each group

(for specific guidance see CONSORT for harms)

Does your paper address CONSORT subitem 19? *

Copy and paste relevant sections from the manuscript (include quotes in quotation marks "like this" to indicate direct quotes from your manuscript), or elaborate on this item by providing additional information not in the ms, or briefly explain why the item is not applicable/relevant for your study

No important harms or unintended effects were reported in this study. Full details regarding the monitoring and reporting of harms or unintended effects for each group are provided in the published manuscript: https://doi.org/10.1093/pm/pnaf035

19-i) Include privacy breaches, technical problems

Include privacy breaches, technical problems. This does not only include physical "harm" to participants, but also incidents such as perceived or real privacy breaches [1], technical problems, and other unexpected/unintended incidents. "Unintended effects" also includes unintended positive effects [2].

	1	2	3	4	5	
subitem not at all important	○	◉	○	○	○	essential

Clear selection

Does your paper address subitem 19-i?

Copy and paste relevant sections from the manuscript (include quotes in quotation marks "like this" to indicate direct quotes from your manuscript), or elaborate on this item by providing additional information not in the ms, or briefly explain why the item is not applicable/relevant for your study

No privacy breaches, technical problems, or other unintended effects (either negative or positive) were reported during the study.

19-ii) Include qualitative feedback from participants or observations from staff/researchers

Include qualitative feedback from participants or observations from staff/researchers, if available, on strengths and shortcomings of the application, especially if they point to unintended/unexpected effects or uses. This includes (if available) reasons for why people did or did not use the application as intended by the developers.

	1	2	3	4	5	
subitem not at all important	○	◉	○	○	○	essential

Clear selection

Does your paper address subitem 19-ii?

Copy and paste relevant sections from the manuscript (include quotes in quotation marks "like this" to indicate direct quotes from your manuscript), or elaborate on this item by providing additional information not in the ms, or briefly explain why the item is not applicable/relevant for your study

Qualitative feedback regarding participants' experiences with the mobile application was collected and has been published separately. Readers are referred to the related publication for detailed findings on user feedback. https://doi.org/10.1177/2164956120987531

DISCUSSION

22) Interpretation consistent with results, balancing benefits and harms, and considering other relevant evidence

NPT: In addition, take into account the choice of the comparator, lack of or partial blinding, and unequal expertise of care providers or centers in each group

22-i) Restate study questions and summarize the answers suggested by the data, starting with primary outcomes and process outcomes (use)

Restate study questions and summarize the answers suggested by the data, starting with primary outcomes and process outcomes (use).

	1	2	3	4	5	
subitem not at all important	○	○	○	◉	○	essential

Clear selection

Does your paper address subitem 22-i? *

Copy and paste relevant sections from the manuscript (include quotes in quotation marks "like this" to indicate direct quotes from your manuscript), or elaborate on this item by providing additional information not in the ms, or briefly explain why the item is not applicable/relevant for your study

"This study evaluated the effectiveness of APA, applied at least three times daily for three minutes per session, using smartphone-based EMA to capture real-time pain outcomes in a 3-arm RCT. A three-arms included targeted APA (T-APA), non-targeted APA (NT-APA), and education control groups. This study provides evidence supporting the effectiveness of APA in reducing pain intensity and improving daily functioning and quality of life among older adults with cLBP. Both the T-APA and NT-APA groups demonstrated significant improvements in pain intensity and pain interference assessed by EMA compared to the control group, which underscore the potential of APA as a non-pharmacological intervention for chronic pain management. In addition, the study highlights the advantages of using EMA to address recall bias in pain reporting. Compared to traditional 7-day recall methods, EMA showed lower pain intensity but higher pain interference, offering a better understanding of how pain fluctuates in real time and impacts daily activities."

22-ii) Highlight unanswered new questions, suggest future research

Highlight unanswered new questions, suggest future research.

	1	2	3	4	5	
subitem not at all important	○	○	○	○	◉	essential

Clear selection

Does your paper address subitem 22-ii?

Copy and paste relevant sections from the manuscript (include quotes in quotation marks "like this" to indicate direct quotes from your manuscript), or elaborate on this item by providing additional information not in the ms, or briefly explain why the item is not applicable/relevant for your study

Yes, our manuscript highlights unanswered questions and suggests areas for future research.

Specifically, we note the need for:
"Future studies should focus on optimizing APA regimens, including investigating the ideal duration and frequency of pressing to maximize pain relief without causing potential sensitization."

"Future studies should address the limitations of compliance and retention when using EMA, particularly among older adults with chronic pain, by developing adaptive EMA protocols and participant-centered engagement strategies."

"Further investigation into the biological mechanisms underlying differing responses to APA is necessary to enhance our understanding and improve treatment strategies."

"The study's four-week duration may not have been sufficient to fully assess the long-term impact of APA on pain relief and functional improvements. Future research should consider extended intervention periods using EMA burst designs."

20) Trial limitations, addressing sources of potential bias, imprecision, and, if relevant, multiplicity of analyses

20-i) Typical limitations in ehealth trials

Typical limitations in ehealth trials: Participants in ehealth trials are rarely blinded. Ehealth trials often look at a multiplicity of outcomes, increasing risk for a Type I error. Discuss biases due to non-use of the intervention/usability issues, biases through informed consent procedures, unexpected events.

	1	2	3	4	5	
subitem not at all important	○	○	○	○	●	essential

Clear selection

Does your paper address subitem 20-i? *

Copy and paste relevant sections from the manuscript (include quotes in quotation marks "like this" to indicate direct quotes from your manuscript), or elaborate on this item by providing additional information not in the ms, or briefly explain why the item is not applicable/relevant for your study

Yes, our manuscript discusses several important trial limitations, potential sources of bias, and issues commonly encountered in eHealth trials.

Potential biases:

Selection bias: "Participants without EMA were significantly older... suggesting selection bias in EMA analyses." (Results – Demographics and Discussion – Limitations)

Self-report bias: Pain outcomes were self-reported, which can be influenced by individual perceptions despite the real-time capture method.

Compliance bias: Older participants had lower EMA compliance, possibly affecting the representativeness of longitudinal data.

21) Generalisability (external validity, applicability) of the trial findings

NPT: External validity of the trial findings according to the intervention, comparators, patients, and care providers or centers involved in the trial

21-i) Generalizability to other populations

Generalizability to other populations: In particular, discuss generalizability to a general Internet population, outside of a RCT setting, and general patient population, including applicability of the study results for other organizations

	1	2	3	4	5	
subitem not at all important	○	○	○	◉	○	essential

Clear selection

Does your paper address subitem 21-i?

Copy and paste relevant sections from the manuscript (include quotes in quotation marks "like this" to indicate direct quotes from your manuscript), or elaborate on this item by providing additional information not in the ms, or briefly explain why the item is not applicable/relevant for your study

"Despite its contributions, this study had limitations that warrant consideration. The relatively low EMA compliance rate (44.8%) and high attrition rate (54%) may have affected the completeness of real-time data collection, limiting the generalizability of findings. Older participants showed lower EMA compliance, suggesting potential barriers such as fatigue, cognitive burden, or difficulty integrating frequent assessments into daily routines. Addressing these challenges in future research—such as through adaptive EMA protocols or participant-centered engagement strategies—will be critical for improving retention and data reliability."

21-ii) Discuss if there were elements in the RCT that would be different in a routine application setting

Discuss if there were elements in the RCT that would be different in a routine application setting (e.g., prompts/reminders, more human involvement, training sessions or other co-interventions) and what impact the omission of these elements could have on use, adoption, or outcomes if the intervention is applied outside of a RCT setting.

	1	2	3	4	5	
subitem not at all important	○	○	○	○	◉	essential

Clear selection

Does your paper address subitem 21-ii?

Copy and paste relevant sections from the manuscript (include quotes in quotation marks "like this" to indicate direct quotes from your manuscript), or elaborate on this item by providing additional information not in the ms, or briefly explain why the item is not applicable/relevant for your study

"In adapting this intervention to routine practice, the role of EMA would need to be carefully reconsidered, as several supportive elements from the RCT would likely be absent. In the RCT, participants received automated prompts and follow-up calls to maintain EMA compliance, along with in-person training and weekly reinforcement, which would be difficult to replicate in real-world settings. Without these supports, lower compliance and incomplete EMA data are likely. Participants also used standardized smartphones with a simplified app, whereas in practice, variability in devices and digital literacy could increase technical challenges. Future adaptation should include automated reminders, user-friendly designs, built-in troubleshooting, and brief onboarding sessions to sustain engagement. Without such strategies, lower EMA completion rates could limit the ability to personalize and optimize interventions like APA in routine care."

OTHER INFORMATION

23) Registration number and name of trial registry

Does your paper address CONSORT subitem 23? *

Copy and paste relevant sections from the manuscript (include quotes in quotation marks "like this" to indicate direct quotes from your manuscript), or elaborate on this item by providing additional information not in the ms, or briefly explain why the item is not applicable/relevant for your study

"The study protocol was published [7] and registered in the US Clinical Trials Registry (Trial ID: NCT03589703). This study was approved by the Johns Hopkins Medicine Institutional Review Board (IRB00175409)"

24) Where the full trial protocol can be accessed, if available

Does your paper address CONSORT subitem 24? *

Cite a Multimedia Appendix, other reference, or copy and paste relevant sections from the manuscript (include quotes in quotation marks "like this" to indicate direct quotes from your manuscript), or elaborate on this item by providing additional information not in the ms, or briefly explain why the item is not applicable/relevant for your study

"The study protocol was published [7] and registered in the US Clinical Trials Registry (Trial ID: NCT03589703)." DOI: 10.1186/s13063-019-4016-x

25) Sources of funding and other support (such as supply of drugs), role of funders

Does your paper address CONSORT subitem 25? *

Copy and paste relevant sections from the manuscript (include quotes in quotation marks "like this" to indicate direct quotes from your manuscript), or elaborate on this item by providing additional information not in the ms, or briefly explain why the item is not applicable/relevant for your study

"This analysis is part of a study supported by the National Institute of Aging (R01AG056587)"

X27) Conflicts of Interest (not a CONSORT item)

X27-i) State the relation of the study team towards the system being evaluated

In addition to the usual declaration of interests (financial or otherwise), also state the relation of the study team towards the system being evaluated, i.e., state if the authors/evaluators are distinct from or identical with the developers/sponsors of the intervention.

	1	2	3	4	5	
subitem not at all important	○	○	○	○	●	essential

Clear selection

Does your paper address subitem X27-i?

Copy and paste relevant sections from the manuscript (include quotes in quotation marks "like this" to indicate direct quotes from your manuscript), or elaborate on this item by providing additional information not in the ms, or briefly explain why the item is not applicable/relevant for your study

"All authors have no conflicts of interest to declare."

About the CONSORT EHEALTH checklist

As a result of using this checklist, did you make changes in your manuscript? *

- ○ yes, major changes
- ◉ yes, minor changes
- ○ no

What were the most important changes you made as a result of using this checklist?

The most important change was providing greater detail in the Methods section to ensure clarity and completeness for readers. Since this paper presents secondary analyses from a previously published RCT, including its main outcomes, protocol, and app development, additional descriptions were necessary to make this manuscript self-contained.

How much time did you spend on going through the checklist INCLUDING making changes in your manuscript *

Approximately 10 to 12 hours were spent reviewing the checklist and making the necessary changes to the manuscript.

As a result of using this checklist, do you think your manuscript has improved? *

- [x] yes
- [ ] no
- [ ] Other:

Would you like to become involved in the CONSORT EHEALTH group?

This would involve for example becoming involved in participating in a workshop and writing an "Explanation and Elaboration" document

- [x] yes
- [ ] no
- [ ] Other:

Clear selection

Any other comments or questions on CONSORT EHEALTH

Please remove the requirement for a minimum of 25 characters from the following question: "How much time did you spend going through the checklist, including making changes to your manuscript?"

STOP - Save this form as PDF before you click submit

To generate a record that you filled in this form, we recommend to generate a PDF of this page (on a Mac, simply select "print" and then select "print as PDF") before you submit it.

When you submit your (revised) paper to JMIR, please upload the PDF as supplementary file.

Don't worry if some text in the textboxes is cut off, as we still have the complete information in our database. Thank you!

Final step: Click submit !

Click submit so we have your answers in our database!

Submit Clear form

Never submit passwords through Google Forms.

This content is neither created nor endorsed by Google. - Terms of Service - Privacy Policy

Does this form look suspicious? Report

Google Forms
