# Supplementary material for: Smartphone-Based Ecological Momentary Assessment of Pain in Older Adults Undergoing Auricular Point Acupressure for Chronic Low Back Pain: Secondary Analysis of a Randomized Controlled Trial
Source: JMIR Form Res. 2026 Mar 4;10:e79612. doi: 10.2196/79612 (PMC13000383; doi:10.2196/79612)
Supplement: Multimedia Appendix 2 [file formative_v10i1e79612_app2.docx]

**Multimedia Appendix 2**

Table 1. Demographics and baseline characteristics by EMA availability (N = 272)

| **Characteristic** | **With EMA (n=211)** | **Without EMA (n=61)** | **Overall (N=272)** | ***P*-value** |
| --- | --- | --- | --- | --- |
| **Age (years), Mean (SD)** | 69.36 (6.70) | 72.15 (7.41) | 69.99 (6.95) | 0.006* |
| **Gender, n (%)** |  |  |  | 0.744 |
| Female | 135 (64.0%) | 39 (63.9%) | 174 (64.0%) |  |
| Male | 74 (35.1%) | 22 (36.1%) | 96 (35.3%) |  |
| Unspecified | 2 (0.9%) | 0 (0%) | 2 (0.7%) |  |
| **Race, n (%)** |  |  |  | 0.291 |
| American Indian or Alaska Native | 2 (0.95%) | 0 (0%) | 2 (0.74%) |  |
| Asian | 1 (0.47%) | 0 (0%) | 1 (0.37%) |  |
| Black or African American | 134 (63.5%) | 30 (49.2%) | 164 (60.3%) |  |
| White | 68 (32.2%) | 29 (47.5%) | 97 (35.7%) |  |
| Native Hawaiian/Pacific Islander | 1 (0.47%) | 0 (0%) | 1 (0.37%) |  |
| More than one race | 3 (1.42%) | 0 (0%) | 3 (1.10%) |  |
| Unknown or Not Reported | 2 (0.95%) | 2 (3.28%) | 4 (1.48%) |  |
| **Ethnicity, n (%)** |  |  |  | 0.770 |
| Hispanic | 3 (1.4%) | 1 (1.6%) | 4 (1.5%) |  |
| Not Hispanic | 172 (81.5%) | 46 (75.4%) | 218 (80.2%) |  |
| Unknown | 36 (17.1%) | 14 (23.0%) | 50 (18.3%) |  |
| **BMI, mean (SD)** | 30.89 (7.56) | 31.18 (8.32) | 30.95 (7.71) | 0.800 |
| **Education level, n (%)** |  |  |  | 0.633 |
| High school or less | 74 (26.5%) | 24 (33.3%) | 76 (27.2%) |  |
| College or higher | 98 (46.5%) | 30 (41.7%) | 107 (39.3%) |  |
| Other/Unknown | 39 (27.0%) | 7 (25.0%) | 89 (33.5%) |  |
| **Employment status, n (%)** |  |  |  | 0.064 |
| Working | 26 (12.3%) | 6 (9.8%) | 32 (11.8%) |  |
| Retired | 117 (55.5%) | 45 (73.8%) | 162 (59.6%) |  |
| Other | 68 (32.2%) | 10 (16.4%) | 78 (28.7%) |  |
| **Smoking status, n (%)** |  |  |  | 0.094 |
| Current smoker | 42 (19.9%) | 5 (8.2%) | 47 (17.3%) |  |
| Never smoked | 76 (36.0%) | 27 (44.3%) | 103 (37.9%) |  |
| Previously smoked | 93 (44.1%) | 29 (47.5%) | 122 (44.8%) |  |
| **Opioid use, n (%)** |  |  |  | 0.246 |
| Yes | 97 (46.0%) | (39.3%) | 121 (44.5%) |  |
| No | 103 (48.8%) | (59.0%) | 139 (51.1%) |  |
| Not sure | 11 (5.2%) | 1 (1.6%) | 12 (4.4%) |  |
| **Baseline pain and pain interference, mean (SD)** |  |  |  |  |
| Worst pain | 7.17 (1.68) | 7.40 (1.84) | 7.22 (1.71) | 0.361 |
| Average pain | 6.05 (1.82) | 6.07 (1.86) | 6.06 (1.82) | 0.960 |
| Current pain | 4.67 (2.76) | 4.56 (2.62) | 4.64 (2.73) | 0.780 |
| Pain interference with enjoyment of life | 4.74 (3.00) | 3.80 (3.42) | 4.53 (3.12) | 0.041 |
| Pain interference with general activity | 5.20 (2.49) | 4.51 (3.05) | 5.05 (2.63) | 0.069 |
| **Group, n (%)** |  |  |  | 0.404 |
| T-APA | 72 (34.1%) | 20 (32.8%) | 92 (33.8%) |  |
| NT-APA | 74 (35.1%) | 17 (27.9%) | 91 (33.5%) |  |
| Control | 65 (30.8%) | 24 (39.3%) | 89 (32.7%) |  |

SD, Standard Deviation; BMI, Body Mass Index; T-APA, targeted APA; NT-APA , non-targeted APA; *: p-value < 0.05.
