# Supplementary material for: Smartphone-Based Ecological Momentary Assessment of Pain in Older Adults Undergoing Auricular Point Acupressure for Chronic Low Back Pain: Secondary Analysis of a Randomized Controlled Trial
Source: JMIR Form Res. 2026 Mar 4;10:e79612. doi: 10.2196/79612 (PMC13000383; doi:10.2196/79612)
Supplement: Multimedia Appendix 3 [file formative_v10i1e79612_app3.docx]

**Multimedia Appendix 3**

Distribution of individual ecological momentary assessment compliance rates among participants included in the secondary analysis. Frequency represents the number of participants at each level of EMA compliance.


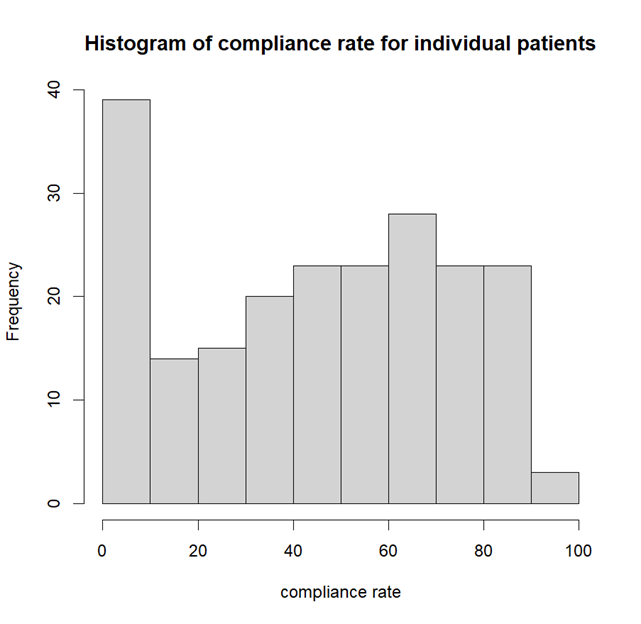


**(n = 211)**
